# Plasma-derived extracellular vesicles prime alveolar macrophages for autophagy and ferroptosis in sepsis-induced acute lung injury

**DOI:** 10.1186/s10020-025-01111-x

**Published:** 2025-02-04

**Authors:** Rongzong Ye, Yating Wei, Jingwen Li, Yu Zhong, Xiukai Chen, Chaoqian Li

**Affiliations:** 1 Department of Emergency Medicine, The First Affiliated Hospital of Guangxi Medical University, Nanning, 530021 China; 2 Guangxi Medical University, Nanning, 530021 China; 3 Department of Critical Care Medicine, The First Affiliated Hospital of Xiamen University, Xiamen, 361000 China

**Keywords:** Extracellular vesicles, MicroRNAs, Sepsis-induced acute lung injury, Alveolar macrophages, Autophagy, Ferroptosis

## Abstract

Sepsis-induced acute respiratory distress syndrome (ARDS) is a severe complication of sepsis and the leading cause of mortality. Although the role of alveolar macrophages (AMs) in stabilizing pulmonary homeostasis is well established, the effects of circulating extracellular vesicles (EVs) on AMs remain largely unknown. In this study, an investigation was conducted to map the miRNA and protein expression profiles of EVs derived from septic plasma. Notably, EV-based panels (miR-122-5p, miR-125b-5p, miR-223-3p, OLFM4, and LCN2) have been found to be associated with the severity or prognosis of sepsis, with promising AUC values. Moreover, the levels of LCN2, miR-122-5p, and miR-223-3p were identified as independent predictors of septic ARDS. The in vitro coculture results revealed that the effects of LPS-EVs from the plasma of sepsis-induced acute lung injury (ALI), which carry pro-inflammatory EVs, were partly mediated by miR-223-3p, as evidenced by the promotion of inflammation, autophagy and ferroptosis in AMs. Mechanistically, the upregulation of miR-223-3p in LPS-EVs triggers autophagy and ferroptosis in AMs by activating Hippo signaling via the targeting of MEF2C. In vivo, the inhibition of miR-223-3p effectively mitigated LPS-EV-induced inflammation and AM death in the lungs, as well as histological lesions. Overall, miR-223-3p in LPS-EVs contributes to sepsis-induced ALI by priming AMs for autophagy and ferroptosis through the MEF2C/Hippo signaling pathway. These findings suggest a novel mechanism of plasma-AM interaction in sepsis-induced ALI, offering a plausible strategy for assessing septic progression and treating lung injury.

**Supplementary Information:**

The online version contains supplementary material available at 10.1186/s10020-025-01111-x.

## Introduction

Sepsis, characterized by a systemic inflammatory response syndrome, has the potential to induce multi-organ dysfunction and escalate to a life-threatening condition (Zhang and Ning 2021; Huang et al. 2019; Xie et al. 2020). Sepsis is the most prevalent extrapulmonary cause of acute respiratory distress syndrome (ARDS), accounting for approximately 32% of cases (Bersten et al. 2002). Recent research suggests that septic ARDS is associated with a more severe clinical course than ARDS caused by other etiologies, resulting in a worse prognosis and higher mortality rates (Zhou et al. 2014). The discovery of new biomarkers for the early prediction of ARDS in sepsis patients is an essential clinical need, as it would improve the triage and treatment of these patients. Nevertheless, reliable and effective methods for the early detection and prognostic prediction of septic ARDS in the clinic are lacking.

Alveolar macrophages (AMs) are the main type of macrophage in the lung, located in the alveolar air-exposed space and closely attached to epithelial cells (Mass et al. 2023). They serve as the primary gatekeepers and caretakers of the alveolus, engulfing cellular and pathogenic debris while removing mucus material from this area, which is crucial for maintaining optimal gas exchange in the lung (Hu et al. 2021). It is widely accepted that AMs serve as the primary initiating cells of the local inflammatory response in the lung. However, in an inflammatory context, AMs produce a significant quantity of inflammatory mediators, leading to the activation of other immune cells and the release of inflammatory factors, ultimately resulting in uncontrolled local inflammation (Matthay and Zimmerman 2005). Therefore, the study of targeting AMs has a broad prospect, and its intervention strategy is also expected to be a novel approach for the treatment of septic ARDS.

Extracellular vesicles (EVs), released from various types of cells, play a crucial role in intercellular communication and regulation, exerting local and systemic effects that impact a range of disease processes (Gao and Raj 2020). Evidence suggests that biomolecules (such as proteins, mRNAs, miRNAs, lncRNAs, DNAs, lipids, and metabolites) expressed in EVs released from damaged or diseased cells can serve as indicators of disease (Das et al. 2024). Inflamed endothelial cells remotely activate neutrophil degranulation by releasing EVs, promoting the reverse transendothelial migration process of PMNs and subsequent remote lung injury (Zi et al. 2024). Another study found that circ_000178 in plasma-derived EVs (plasma-EVs) delays atherosclerosis progression by mitigating endothelial cell injury through the miR-513a-5p/TGFBR3 ceRNA network mechanism (Tong et al. 2023). Hence, circulating EVs hold promise as a novel therapeutic strategy for a range of medical conditions. However, the specific involvement of plasma-EVs in triggering sepsis-induced ALI remains elusive. Additionally, it has been reported that exosomal SCIMP mediates communication between resident macrophages and circulating neutrophils during pneumonia (Pei et al. 2024). Exosomal miR-30d-5p derived from polymorphonuclear neutrophils (PMNs) contributes to sepsis-induced ALI by promoting M1 macrophage polarization and triggering macrophage pyroptosis (Jiao et al. 2021), but the role of PMN-related proteins in the disease is still not understood. Hence, our study sought to elucidate the interaction between plasma-EVs and AMs, potentially offering new insights into the pathogenesis of septic ARDS.

Herein, we investigated the potential of PMN-related proteins and miRNAs in plasma-EVs as biomarkers for septic ARDS. Through analysis and validation of the miRNA and protein expression profiles of EVs derived from septic plasma, upregulated miR-223-3p was identified in septic ARDS. Moreover, the association between miR-223-3p and sepsis-induced ALI was investigated through clinical and in vivo assays. Then, we explored the role of plasma-EVs in promoting autophagy and ferroptosis in AMs. Finally, the underlying mechanisms of the effects of miR-223-3p in plasma-EVs on AM death were elucidated through in vitro and in vivo experiments, revealing previously unrecognized plasma-to-AM communication mediated by EVs.

## Materials and methods

### Patients and control subjects

The protocols, procedures, and recruitment of subjects/patients in this study were approved by the Ethics Committee of the First Affiliated Hospital of Guangxi Medical University, China (Approval No.2023-S492-01), in accordance with the Declaration of Helsinki for biomedical research involving human subjects. Informed consent was obtained from all the subjects or their next of kin prior to enrollment. Blood samples and clinical data were collected from 115 sepsis patients at the Department of Emergency Medicine of the First Affiliated Hospital of Guangxi Medical University for this study. The diagnosis of sepsis followed the criteria outlined in the third International Consensus Definitions for Sepsis and Septic Shock (Sepsis-3) (Singer et al. 2016). Patients with ARDS were enrolled according to the Berlin definition of ARDS (acute hypoxemia, partial pressure of arterial oxygen (PaO_2_)/fraction of inspired oxygen (FiO_2_) ratio < 300 mmHg, bilateral pulmonary infiltrates on chest radiography, and not explained by cardiac edema) (Ranieri et al. 2012). The following individuals were not included in the study: pregnant patients, those with a history of congenital heart disease, coronary heart disease, myocardial infarction, hypertensive heart disease, pulmonary hypertension, chronic heart dysfunction, tumors or organ transplants; individuals with an immunosuppressive or immune-deficient condition; and those who could not be contacted during follow-up. Based on the 28-day prognosis and clinical data of the patients, they were retrospectively diagnosed and grouped for analysis. The study enrolled a total of 157 patients diagnosed with sepsis, among whom 18 met the exclusion criteria after admission and underwent thorough examination, 13 were excluded due to insufficient samples for RT-qPCR or ELISA analysis, and 11 were lost to follow-up, resulting in the final enrollment of 115 patients in the validation cohort. Additionally, 24 healthy donors were enrolled as controls, with an average age of 39 ± 9 years, including 13 males (54.2%) and 11 females (45.8%). We retrospectively collected baseline characteristics, demographic data, routine biochemical parameters, and clinical variables.

### EV purification and characterization

Within 24 h of patient admission, peripheral blood samples were collected from patients in EDTA tubes following a standard venipuncture procedure. Samples need to be processed promptly within 2 h. The collected blood was pretreated and stored at -80℃after the following steps:1500 × g for 20 min at 4℃; 3000 × g for 15 min at 4℃. The isolation of EVs via ultracentrifugation (UC) followed by size exclusion chromatography (SEC) was optimized according to the previously described approach (Li et al. 2022; Wei et al. 2020). Briefly, after being thawed at 37℃, the supernatant was diluted with a seven-fold volume of phosphate-buffered saline (PBS), then centrifuged at 13,000 × g for 30 min and filtered through a 0.22 μm filter to remove large particles. The supernatant of plasma was then subjected to UC using a Type70Ti (Beckman, USA) at 100,000 × g for 2 h at 4℃ to pellet the EVs. The pellet was re-suspended in PBS and centrifuged again at 100,000 × g for 2 h at 4℃. After washing with PBS, the EVs pellet was re-suspended in 1mL PBS. The SEC method utilizing an Exosupur Exclusion Column (Echobiotech, China) as per manufacturer’s instructions was employed to separate the EVs. The sample was eluted with PBS, and 2mL of the target fraction was collected. Subsequently, the EVs solution underwent concentration using an Amicon^®^ ultrafiltration tube with a molecular weight cutoff of 100 kDa (Merck, Germany).

After isolation, the EVs were characterized using Nano flow cytometry (NanoFCM) and transmission electron microscopy (TEM). Western blotting analysis was employed to detect well-established EV biomarkers (Alix, Tsg101, and CD9) as well as non-EV markers (GM130, Calnexin, and TIM23).

### Real-time quantitative polymerase chain reaction (RT-qPCR)

Total RNA was extracted from plasma-EVs using an Exosome RNA Purification Kit (5202050, Simgen). The cells were also subjected to total RNA isolation using RNAiso Plus (TaKaRa, Dalian, China).The Mir-X™ miRNA First-Strand Synthesis Kit (TaKaRa, Dalian, China) and Reverse Transcription Kit (TaKaRa, Dalian, China) were utilized for reverse transcription of miRNAs and mRNAs respectively. PCR analysis was conducted with TB Green Advantage qPCR Premix (TaKaRa, Dalian, China) on ABI-7500 Real-Time PCR Detection System (Applied Biosystems). The levels of miRNA and mRNA were normalized to U6 small nuclear RNA and β-actin as endogenous controls. The relative expression of these RNAs was calculated using the 2^–∆∆CT^ method. The primer sequence can be found in Table S1.

### Enzyme-linked immunosorbent assay (ELISA)

EVs were lysed with RIPA buffer, and the loading amount was adjusted to 100 µL with the sample buffer. Quantification of PMN-related proteins in the plasma-EVs was determined by SLPI (Cloud-Clone Corp, China), OLFM4 (ELK Biotechnology, China), LCN2 (ELK Biotechnology, China), and CD177 (ELK Biotechnology, China). The levels of interleukin-6 (IL-6), tumor necrosis factor-α (TNF-α) and interleukin-1β (IL-1β) in the serum or BALF samples were assessed using ELISA kits from Cloud-Clone Corp., China, following the provided protocols.

### Animals

Male Sprague‒Dawley rats (6–8 weeks old) were obtained from the Animal Centre of Guangxi Medical University. The rats were maintained in a specific pathogen-free environment, and all the experimental procedures were evaluated and approved by the Ethics Committee for Animal Studies at Guangxi Medical University (Approval No.202210303).

### Cell culture

The NR8383 rat alveolar macrophage line was obtained from Pricella Biotechnology (Wuhan, China). The alveolar macrophages were cultured in a humidified environment with 95% air and 5% CO_2_, using Ham’s F-12 K supplemented with 20% FBS and 1% penicillin/streptomycin. Plasm-EVs and AMs were co-cultured in EV-depleted FBS medium.

### In vivo lipopolysaccharide-induced acute lung injury model

Pathogen-free, 8-week-old male Sprague‒Dawley rats were utilized to establish a lipopolysaccharide (LPS)-induced ALI model. Briefly, the rats were anesthetized with isoflurane inhalation and a small incision was made to expose the trachea. Then, 100 µL of LPS (O111:B4, Sigma, USA) at a concentration of 10 mg/kg was slowly injected into the lungs through the distal end of the trachea using a microinjector. The rats in the control group received an equivalent volume of PBS through airway infusion. After 24 h, blood samples were obtained via an EDTA tubes, and the rats were euthanized.

### In vivo injection and in vitro co-culture of EVs

A BCA protein assay kit was used to determine protein concentrations. In vivo, rats in each group were given injections of PBS-EVs or LPS-EVs (300 µg/rat) through the trachea. Twenty-four hours after the administration, the rats were euthanized, and lung tissues and bronchoalveolar lavage fluid (BALF) were collected for analysis. An equal number of rats in the control group received injections of PBS. In vitro, AMs were co-cultured with PBS-EVs or LPS-EVs (100 µg/mL) at 37 °C for 24 h.

### Histopathology

The lungs of individual rats were extracted and immediately fixed in 4% paraformaldehyde, followed by hematoxylin and eosin (H&E) staining. Subsequently, at least ten random microscopic field images were captured from each rat slide to evaluate the severity of ALI on the basis of the level of inflammatory cell infiltration and thickening of the alveolar walls.

### Pulmonary wet-to-dry weight (W/D) ratio

The right upper lung lobes were removed from the rats in each group, and any residual moisture and blood on the surface were absorbed with paper towels. The lungs were measured via an electronic scale and then placed in an oven at 80 °C for 48 h until they reached a constant weight. The dry weight was measured and utilized to calculate the lung W/D ratio.

### Pulmonary microvascular leakage test

Evans blue staining involved injecting 2 mL/kg of Evans blue (0.5%) into the tail vein 30 min before sampling, followed by rinsing the pulmonary circulation with 20 mL of PBS. The lung tissues were then promptly rinsed with PBS and flash-frozen in liquid nitrogen after excision. The homogenized lung tissues were incubated with formamide at 60 °C for 36 h, followed by centrifugation at 5000 × g for 30 min to collect the supernatant. The absorbance (A630) was measured using a multifunctional microplate reader (BioTek) to determine the Evans blue levels in the tissues.

### Western blotting

EVs, cells, and lung tissue were lysed using RIPA lysis buffer. Protein extraction and western blotting were performed as previously reported (Ge et al. 2021). The membranes were visualized using anti-rabbit IgG (H + L) (DyLight™ 800 4X PEG Conjugate) (Cell Signaling Technology, USA). Images were captured with Odyssey software and quantified with ImageJ analytical software on the basis of optical density. The utilized antibodies and their respective dilutions are detailed in Table S2.

### Immunofluorescence staining

Immunofluorescence staining of the cells was performed as previously described (Gong et al. 2022). The samples were observed under an Olympus fluorescence microscope (Olympus, Japan).

### EV labelling

EVs were stained with PKH67 (BestBio, China) following the manufacturer’s protocol. The staining process was halted by the addition of EV-depleted FBS. Unbound dye was removed by UC and SEC, and EVs were collected. After co-culturing for 6 h, AMs were stained with DAPI and observed using an Olympus fluorescence microscope (Olympus, Japan).

### Bioinformatics analysis

The method and software for predicting target genes involved the integration of prediction results from TargetScan, miRDB, miRTarBase, StarBase, and TarBase. Gene functions and signaling pathways were annotated using the Gene Ontology (GO) and Kyoto Encyclopedia of Genes and Genomes (KEGG) databases. The TRRUST database was then utilized to predict TF-mRNA relations. A network involving TFs, miRNAs, and mRNAs was subsequently constructed on the basis of their regulatory relationships and visualized using Cytoscape (version 3.10).

### Dualluciferase reporter assay

We constructed plasmid vectors containing both wild-type and mutant versions of the MEF2C 3ʹ UTR with predicted binding sites for miR-223-3p. These constructs were then transfected into HEK293T cells, along with a renilla luciferase vector, in all the transfections to monitor the transfection efficiency. The activities of firefly luciferase were normalized to those of Renilla luciferase.

### In vitro miRNA transfection

Following the protocol of the RNATransMate Kit (Sangon Biotech, China), cells in the exponential growth phase were plated in 60 mm dishes and incubated at 37 °C with 5% CO_2_. When the cells reached 60–70% confluence, each plate was treated with 150 pmol of miR-223-3p mimic, miR-223-3p inhibitor, mimic, negative control (NC), or inhibitor-NC (Sangon Biotech, China) along with 10 µL of RNATransMate.

### In vivo miRNA administration

The antagomir-223-3p and miRNA antago NC were produced by Sangon Biotech, Shanghai, China. Each group of male rats received an intravenous injection of 100 nmol of antagomir-223-3p or a negative control one day prior to the injection of LPS-EVs. Tissue samples were collected for further analysis 24 h after the injections.

### Statistical analysis

Statistical analyses were conducted using SPSS version 26.0 (IBM), and Prism version 9.0 (GraphPad). Student’s t-test was utilized for comparing two groups, while one-way analysis of variance (ANOVA) followed by Tukey’s multiple comparisons test was used for multiple group comparisons. Spearman correlation was employed for correlation analysis. Hazard ratios and accompanying 95% confidence intervals were computed using a logistic regression model. The diagnostic/prognosis performance of the target miRNAs/proteins was evaluated using receiver operating characteristic curve (ROC) analysis. To illustrate the impact graphically, the data on miRNA or protein expression were dichotomized on the basis of low versus high expression at the median. Kaplan-Meier analysis was used to assess overall survival. *P* < 0.05 was considered statistically significant.

## Results

### Variation in miRNAs and proteins in plasma-EVs between sepsis patients with and without ARDS

**Fig. 1:**
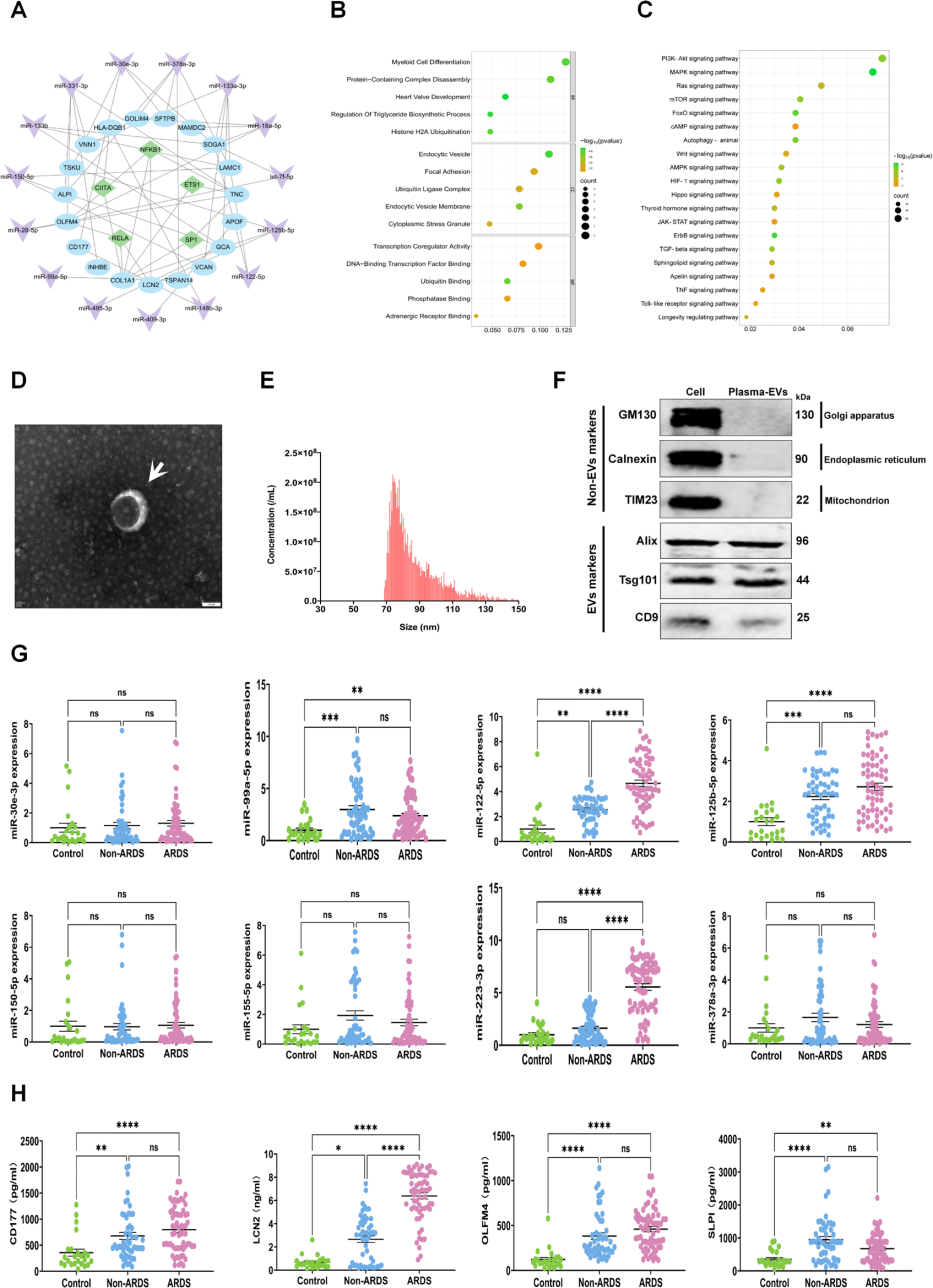
miRNA and protein analysis of plasma-EVs. **(A)** TF-miRNA-mRNA regulatory networks were constructed for sepsis based on their inside interaction relationships. **(B)** The GO enrichment analysis of the target genes of the eight upregulated miRNAs. **(C)** The KEGG enrichment analysis of the target genes of the eight upregulated miRNAs. **(D)** Observation of EV morphology by TEM; Scale bar: 100 nm. **(E)** NanoFCM of EV diameter. **(F)** Western blot analysis of the expression of EV markers (Alix, Tsg101, and CD9) and Non-EV markers (GM130, calnexin, and TIM23) in cells and EVs. **(G)** Validation of the expression levels of miR-30e-3p, miR-99a-5p, miR-122-5p, miR-125b-5p, miR-150-5p, miR-155-5p, miR-223-3p, and miR-378a-3p in plasma-EVs by RT-qPCR. **(H)** Validation of the expression levels of PMN-related proteins (CD177, SLPI, OLFM4 and LCN2) in plasma-EVs by ELISA. Data are presented as the mean ± SEM. ns, no significance, *p < 0.05, **p < 0.01, ***p < 0.001,****p < 0.0001

Given that miRNAs and proteins in EVs provide a means of assessing physiological and pathological status, it is imperative to investigate their potential involvement in the early prediction or assessment of sepsis progression. In this study, we investigated several miRNAs and proteins associated with sepsis in plasma-EVs, as reported in previous studies (Gong et al. 2022; Shin et al. 2023). We utilized the miRWalk database to predict the target genes of 21 DE-miRNAs, which collectively corresponded to the mRNAs of 40 proteins present in EVs. A TF-miRNA-mRNA regulatory network involving 5 transcription factors, 15 miRNAs, and 19 target genes was constructed (Fig. 1A). We subsequently used the TargetScan database to predict the target genes of the 8 upregulated DE-miRNAs, which collectively encompassed 2523 target genes. GO analysis revealed 2094 significantly enriched GO terms, including 1709 biological processes, 157 cellular components and 228 molecular functions involving protein-containing complex disassembly, focal adhesion, and transcription coregulator activity, etc. (Fig. 1B). In addition, 127 significantly enriched pathways, involving mainly the MAPK signaling pathway, the FoxO signaling pathway and autophagy, among others, were identified via KEGG analysis (Fig. 1C). Overall, these results indicate that EVs from plasma, as well as those from different pathological statuses, carry different miRNAs and proteins that may have distinct pathophysiological roles.

EVs were isolated from the plasma of septic patients through ultracentrifugation and molecular size exclusion. Transmission electron microscopy (TEM) images showed that the isolated EVs exhibited a spherical morphology and possessed a bilayered membrane structure (Fig. 1D). NanoFCM analysis indicated that the size distribution of EVs mainly ranged from 65 to 150 nm, with a mean of 86.6 nm (Fig. 1E). Western blot analysis revealed that the isolated EVs expressed positive EV biomarkers (Alix, Tsg101, and CD9), whereas the Golgi (GM130), endoplasmic reticulum (Calnexin) and mitochondrial proteins (TIM23) were exclusively detected in the cell lysates and served as negative markers for the EVs (Fig. 1F). The above analysis confirmed our efficient isolation of plasma-EVs. To investigate the association between plasma-EVs and sepsis-related pathological development, we validated 8 miRNAs using RT-qPCR and 4 PMN-related proteins using commercially available ELISA in plasma-EVs. A total of 51 septic non-ARDS patients, 64 septic ARDS patients, and 24 healthy blood donors were included in the study for comparison. The demographic and clinical characteristics of the septic non-ARDS patients and septic ARDS patients are presented in Table 1. Notably, the levels of miR-122-5p, miR-223-3p, and LCN2 in the plasma-EVs were significantly elevated in the septic ARDS group compared with those in the septic non-ARDS group (*P* < 0.05) (Fig. 1G, H). Taken together, these datasets suggest that the miRNA and protein patterns of plasma-EVs could serve as biomarkers for distinguishing between septic ARDS and septic non-ARDS patients.

**Table 1:** Demographic and clinical details of patients with septic non-ARDS and septic ARDS

Characteristics	Septic non-ARDS *N* = 51	Septic ARDS *N* = 64	*P*-value
**Sociodemographic characteristics**			
Age, years	57.5 (51.3–64.8)	59.5 (45.5–68)	0.481
Male sex, n(%)	36 (70.6)	50 (78.1)	0.355
**Comorbidities**,** n(%)**			
Arterial hypertension	22 (43.1)	21 (32.8)	0.256
Diabetes mellitus	10 (19.6)	8 (12.5)	0.297
Cerebrovascular disease	2 (3.9)	5 (7.8)	0.386
**Source of sepsis**,** n(%)**			
Lung	18 (35.3)	64 (100)	< 0.001
Abdominal	7 (13.7)	13 (20.3)	0.355
Skin and soft tissue	2 (3.9)	5 (7.8)	0.386
Other	27 (52.9)	2 (3.1)	< 0.001
**ICU admission**			
MAP (mmHg)	88 ± 23.3	83.3 ± 23	0.217
WBC (10^9^/L)	12.7 ± 5.4	14.5 ± 7.2	0.140
HGB (g/L)	95 ± 36	102 ± 35	0.292
PLT (10^9^/L)	192.1 (91.8–280.8)	211.5 (108–288)	0.870
PCT (ng/mL)	2.28 (0.39–11.09)	2.23 (0.54–18.73)	0.458
CRP (mg/L)	75 (13.2-132.2)	94.1 (30.8-163.1)	0.126
proBNP (pg/mL)	2084 (315-17241)	2640 (835–8400)	0.512
PT (s)	13 (12–15)	13.5 (12–16)	0.093
APTT ( s)	33 (30–36)	32 (28–36)	0.769
FIB (g/L)	4.5 ± 1.6	4.2 ± 1.7	0.196
TB (umol/L)	14.7 (10.2–20.8)	14.4 (9.2–27.6)	0.541
Albumin (g/L)	33 ± 6.2	30.8 ± 5	0.047
Scr (umol/L)	92.5 (64–225)	133 (93–223)	0.078
Lactate (mmol/L)	1.63 (1.14–2.55)	2.47 (1.5–4.93)	0.003
Use of mechanical ventilation, n(%)	40 (78.4)	52 (81.3)	0.707
Mechanical ventilation duration (days)	1 (0.6–7.7)	3.9 (1–8)	0.153
PaO_2_/FiO_2_ (mmHg)	350 (291.3–452)	184 (115.3–220)	< 0.001
SOFA score	7.5 (4.8–10)	8 (7-10.8)	0.038
APACHE II score	18 (13.8–22.3)	22 (18.3–25)	0.002
**ICU Stay**			
ICU stay (days)	4 (1-9.3)	4.5 (1–11)	0.950
28-day mortality, n (%)	18 (35.3)	37 (57.8)	0.016

**Abreviations**: ARDS, Acute respiratory distress syndrome; APTT, Activated partial thromboplastin time; APACHE, Acute physiology and chronic health evaluation; CRP, C-reactive protein; FIB, Fibrinogen; ICU = Intensive care unit; MAP, mean arterial pressure; PCT, Procalcitonin; PT, Prothrombin time; pro-BNP, pro-brain natriuretic peptide; PaO_2_/FiO_2_, Ratio of partial pressure of arterial oxygen to the fraction of inspired oxygen; TB, Total bilirubin; Scr, Serum creatinine; SOFA, Sequential organ failure assessment score; WBC, White blood cells

### Validation of an EV-based panel for assessing the severity and prognosis of sepsis

The baseline characteristics of the patients with sepsis and septic shock are presented in Table S3. The median age was 58 (47–68) years, and 74.8% were male. Results revealed that the levels of miR-99a-5p, miR-122-5p, miR-125b-5p, and miR-223-3p were significantly increased in sepsis patients compared with healthy controls. In addition, the levels of miR-122-5p, miR-125b-5p, and miR-223-3p were significantly greater in septic shock patients than in healthy controls and patients with sepsis (Fig. S1A). Compared with those in healthy controls, the levels of CD177, SLPI, OLFM4, and LCN2 were elevated in plasma-EVs from patients with sepsis. In addition, the levels of OLFM4 and LCN2 were greater in patients with septic shock than in those with sepsis (Fig. S1B).

**Fig. 2:**
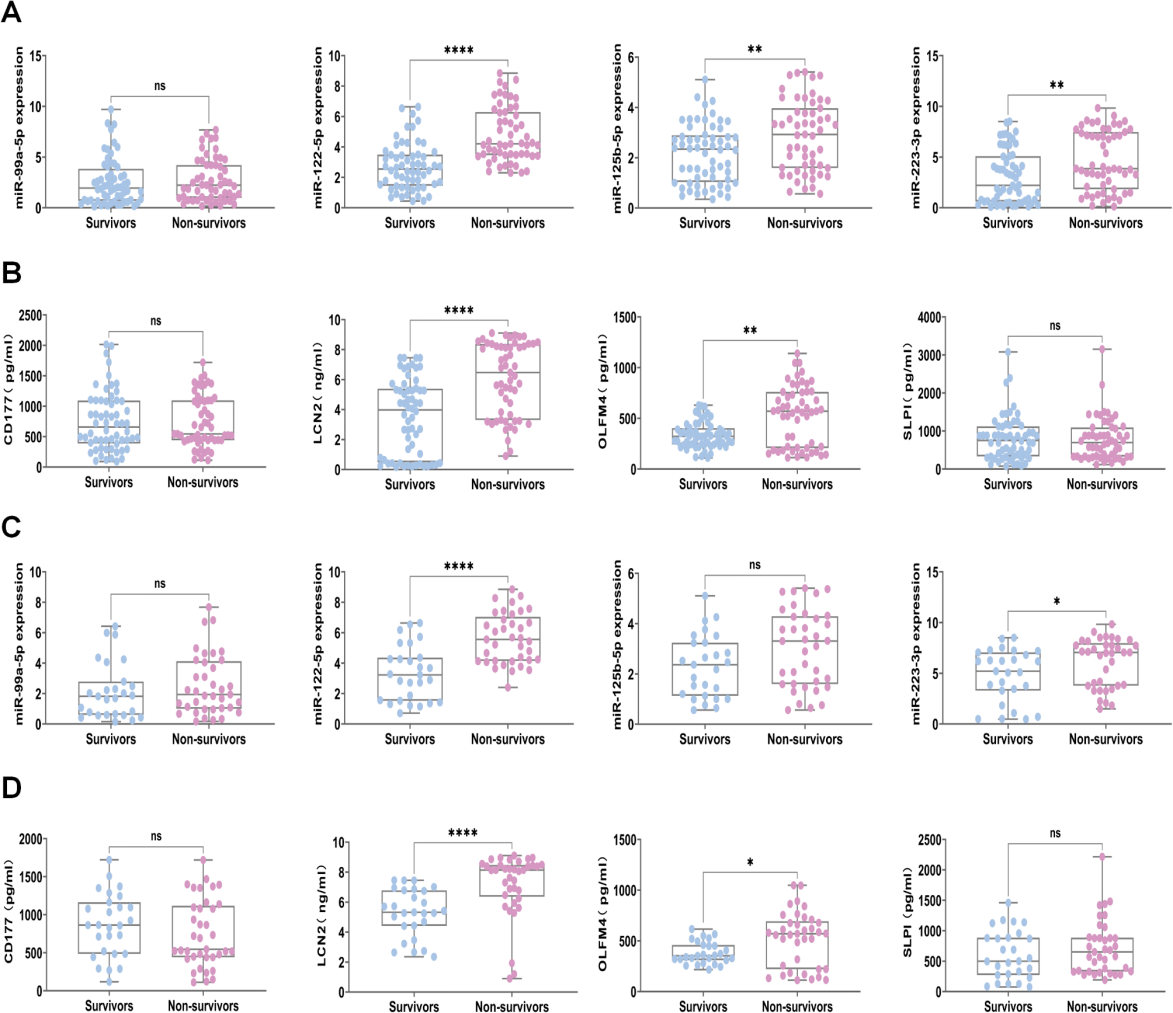
Validation of miRNA and PMN-related protein expression in plasma-EVs from survivors and non-survivors. **(A-B)** Sepsis patients were categorized into survivors (*n* = 60) and non-survivors (*n* = 55) based on 28-day prognosis. Validation of the expression levels of miR-99a-5p, miR-122-5p, miR-125b-5p, miR-223-3p, CD177, SLPI, OLFM4 and LCN2 in plasma-EVs. **(C-D)** A total of 64 septic ARDS were stratified into survivors (*n* = 27) and non-survivors (*n* = 37). Subsets analysis of the levels of miR-99a-5p, miR-122-5p, miR-125b-5p, miR-223-3p, CD177, SLPI, OLFM4 and LCN2 in 28-day prognosis. Data are presented as the mean ± SEM. ns, no significance, *p < 0.05, **p < 0.01, ***p < 0.001,****p < 0.0001

The baseline characteristics of both survivors and non-survivors are presented in Table S4 and Table S5. We further validated these findings in terms of prognosis, and the results revealed that the levels of miR-122-5p, miR-125b-5p, miR-223-3p, OLFM4, and LCN2 in the plasma-EVs of non-survivors were significantly greater than those in those of survivors (Fig. 2A, B). Similarly, verification of potential prognostic biomarkers selected from septic ARDS patients revealed that the expression levels of miR-122-5p, miR-223-3p, OLFM4, and LCN2 were greater in the non-survivor group than in the survivor group (Fig. 2C, D).

### Predictive potential of EV-based panel for septic ARDS

We performed Spearman correlation analysis on the validation sets to assess whether the expression levels of the plasma-EV panel (miR-122-5p, miR-125b-5p, miR-223-3p, OLFM4, and LCN2) were correlated with disease severity. We found that miR-122-5p, LCN2, and OLFM4 expression in plasma-EVs was positively correlated with the SOFA score (Fig. S2A). The expression levels of miR-122-5p, miR-223-3p, LCN2, and OLFM4 were positively correlated with the APACHE II score (Fig. S2B). Notably, the levels of miR-122-5p, miR-223-3p, and LCN2 in the plasma-EVs were inversely correlated with the PaO_2_/FiO_2_ of the patients (Fig. S2C). The levels of miR-223-3p and LCN2 were positively correlated with the duration of mechanical ventilation and were not significantly correlated with the remaining three indicators (Fig. S2D).

Next, we used a logistic regression analysis to determine the best combination of miRNAs and PMN-related proteins to predict septic ARDS, septic shock, and prognosis. The independent predictors of septic ARDS included LCN2 (OR = 1.491, 95% CI 1.073–2.072, *P* = 0.017), miR-122-5p (OR = 1.797, 95% CI 1.081–2.989, *P* = 0.024), and miR-223-3p (OR = 1.767, 95% CI 1.261–2.476, *P* < 0.001) (Table 2). Moreover, OLFM4 (OR = 1.006, 95% CI 1.003–1.010, *P* < 0.001), miR-122-5p (OR = 1.485, 95% CI 1.021–2.158, *P* = 0.038), and miR-125b-5p (OR = 1.729, 95% CI 1.101–2.715, *P* = 0.017) emerged as independent predictors of septic shock (Table S6). Further investigation revealed that independent prognostic factors for 28-day overall survival in patients with sepsis included LCN2 (OR = 1.326, 95% CI 1.019–1.726, *P* = 0.036), OLFM4 (OR = 1.002, 95% CI 1.000-1.005, *P* = 0.023), and miR-122-5p (OR = 1.625, 95% CI 1.137–2.322, *P* = 0.008) (Table S7). Furthermore, high expression of miR-122-5p (OR = 1.886, 95% CI 1.223–2.910, *P* = 0.004) was an independent prognostic factor for sepsis-related ARDS 28-day overall survival (Table S8).

**Table 2:** Logistic regression in patients with septic ARDS

Risk Factors	β	SE	Wald X^2^	*P*	OR	95%CI	
						Lower	Higher
LCN2	0.399	0.168	5.648	0.017	1.491	1.073	2.072
OLFM4	-0.001	0.001	0.625	0.429	0.999	0.996	1.002
miR-122-5p	0.586	0.259	5.108	0.024	1.797	1.081	2.989
miR-125b-5p	-0.337	0.269	1.571	0.210	0.714	0.422	1.209
miR-223-3p	0.569	0.172	10.930	< 0.001	1.767	1.261	2.476

**Abreviations**: CI = confidence interval; OR = odds ratio

**Fig. 3:**
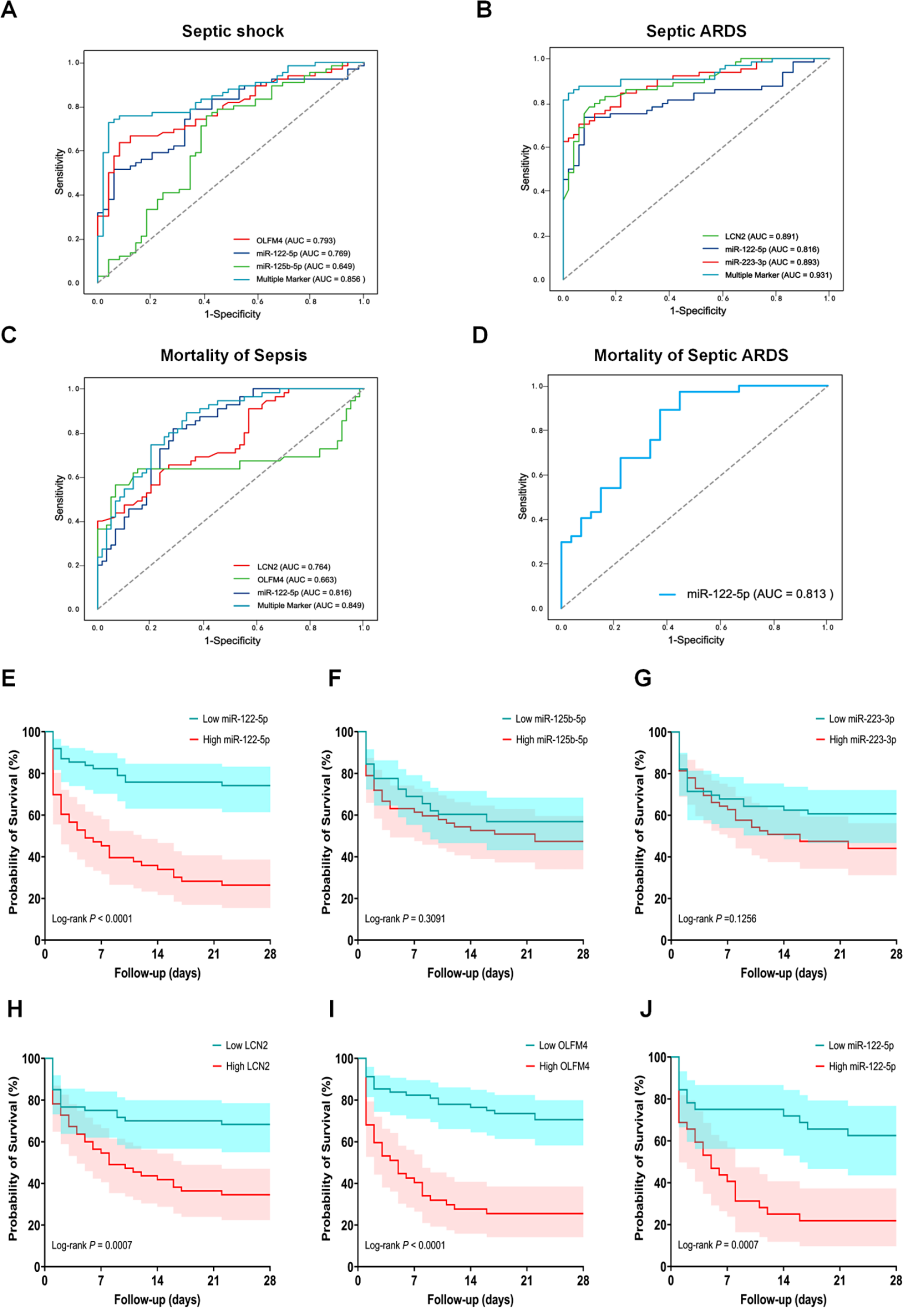
ROC curves and Kaplan-Meier survival estimation of miRNAs and PMN-related proteins from plasma-EVs for septic patients. (**A**-**D**) ROC curves were drawn based on RT-qPCR and ELISA data for septic shock, septic ARDS, and 28-day mortality in septic patients or septic ARDS. AUC indicates area under curve. (**E**-**J**) Kaplan-Meier survival curves according to different levels of miRNAs and PMN-related proteins from plasma-EVs in sepsis. The miRNAs or proteins were dichotomized at the median based on a low-versus-high expression (E, miR-122-5p; F, miR-125b-5p; G, miR-223-3p; H, LCN2; I, OLFM4; J, miR-122-5p in septic ARDS) for 28-day mortality

The validation of the diagnostic accuracy of an EV-based panel in a real-world clinical setting is essential for the clinical significance of miRNAs and proteins in EVs to advance a liquid biopsy assay for sepsis detection and risk stratification. The 5 prominent indicators (miR-122-5p, miR-125b-5p, miR-223-3p, OLFM4, and LCN2) identified in this study were then used to evaluate their diagnostic performance. When OLFM4, miR-122-5p, and miR-125b-5p were combined, the AUC for distinguishing between sepsis and septic shock exceeded 0.8 (Fig. 3A). Moreover, for septic ARDS prediction, ROC analyses revealed that the AUC values of LCN2, miR-122-5p, miR-223-3p, and multiple markers were 0.891, 0.816, 0.893 and 0.931, respectively (Fig. 3B). Notably, the AUC of multiple markers (0.849) was significantly greater than those of LCN2, OLFM4, and miR-122-5p alone for 28-day mortality in patients with sepsis (Fig. 3C). Furthermore, miR-122-5p exhibited the best performance for 28-day mortality in patients with septic ARDS, with an AUC value of 0.813 (Fig. 3D).

Next, the effects of miRNAs and PMN-related proteins from plasma-EVs on the prognosis of patients with sepsis were analyzed. Kaplan-Meier survival analysis revealed that individuals with high expression levels of miR-122-5p, OLFM4, and LCN2 had a worse prognosis than those with low expression levels (Fig. 3E-I). Furthermore, Kaplan-Meier survival analysis revealed a significant difference in 28-day survival between septic ARDS patients with high and low miR-122-5p levels (Fig. 3J). The data indicated that high expression of miR-122-5p, OLFM4, and LCN2 was associated with a low 28-day survival rate in septic patients with poor prognosis, further suggesting that these miRNAs and proteins in EVs have potential in the diagnosis and prognosis of sepsis. We then constructed a regulatory network diagram to illustrate the interactions between the DE-miRNAs and PMN-related proteins in the plasma-EVs. The intersection of the predicted target genes of miR-122-5p, miR-125b-5p, and miR-223-3p from three databases (TargetScan, miRDB, and miRTarBase). TF-miRNA-mRNA regulatory networks, including their internal interaction connections, were established for sepsis via the aforementioned analysis (Fig. S3A, B).

### Plasma-derived EVs promote autophagy and ferroptosis in alveolar macrophages in vitro

Rats were subjected to intratracheal instillation of LPS to generate a model of sepsis-induced ALI. Twenty-four hours following the injection of LPS, characteristic histological damage in the lung, including inflammation, an elevated lung W/D weight ratio, and a relatively high protein concentration in the BALF, was detected. In the serum tests, as determined by several indices (IL-1β, IL-6, and TNF-α), significant differences were observed between the PBS group and the LPS group (Fig. S4A-F). Together, these data suggest that a sepsis-induced ALI model was successfully established in rats. We isolated EVs from the plasma of rats via ultracentrifugation and molecular size exclusion, and the TEM images clearly revealed cup-shaped and lipid bilayer structures of the EVs (Fig. S4G). NanoFCM analysis indicated that the size distribution of EVs mainly ranged from 65 to 150 nm, with a mean of 92.4 nm (Fig. S4H). Western blot analysis revealed that the isolated pellet exhibited positive expression of EV biomarkers (Alix, Tsg101, and CD9), whereas GM130, calnexin, and TIM23 were absent, indicating minimal contamination from the cellular lysate and a high concentration of pure EVs (Fig. S4I). These results demonstrated that the characteristics of plasma-secreted EVs are consistent with those of exosomes.

**Fig. 4:**
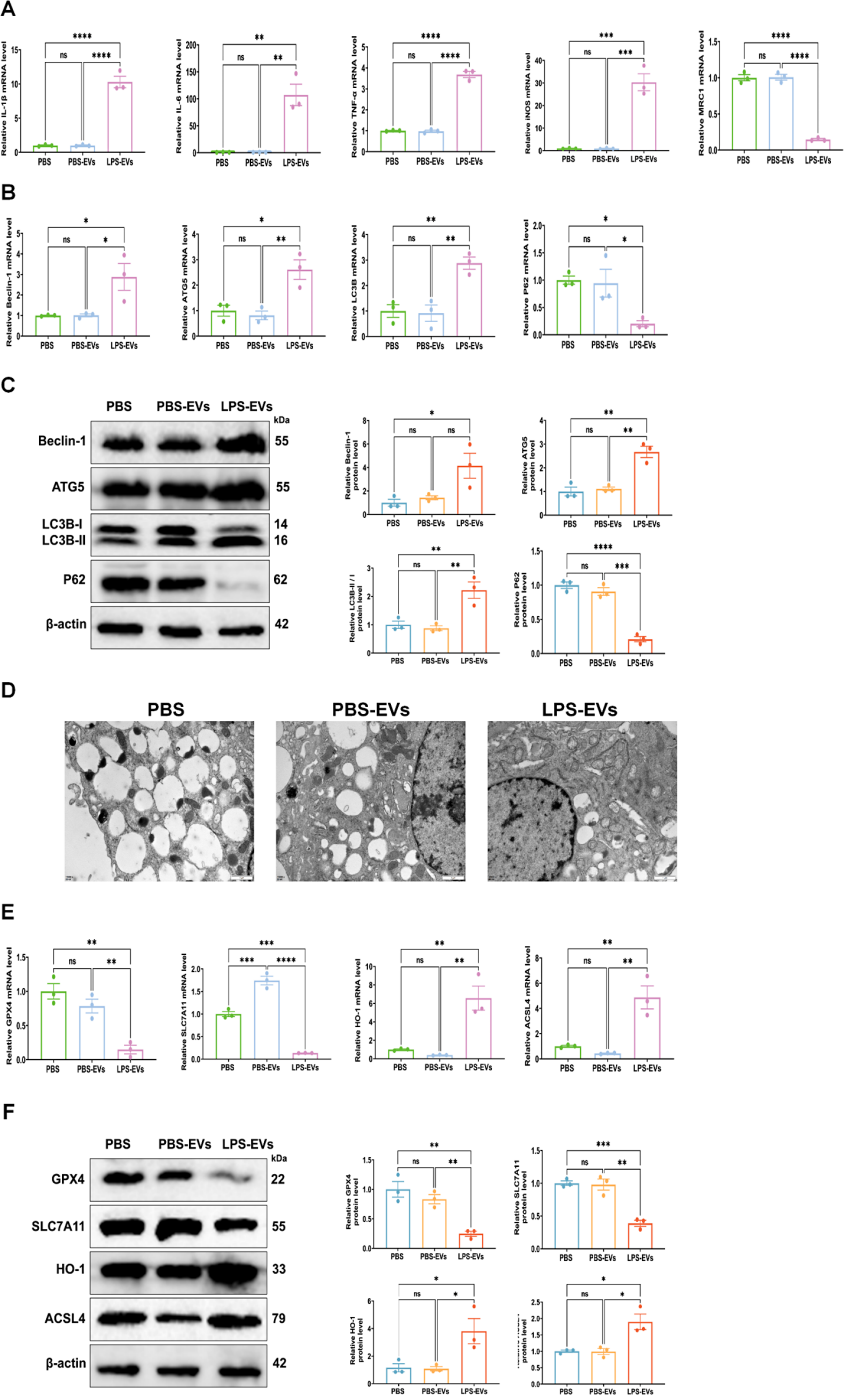
LPS-EVs promoted autophagy and ferroptosis in AMs. **(A)** The mRNA expressions levels of IL-1β, IL-6, TNF-α, iNOS and MRC1 in AMs were analysed by RT-qPCR. **(B)** The mRNA expression of Beclin-1, ATG5, LC3B, and P62 in AMs were analysed by RT-qPCR. **(C)** Western blot analysis of Beclin-1, ATG5, LC3B-II/I, and P62 levels in AMs. The histogram showed the relative ratio of the expression levels of the target protein to that of an internal reference. **(D)** Mitochondrial damage and autophago*s*omes were detected by TEM. **(E)** The mRNA expressions levels of GPX4, SLC7A11, HO-1,and ACSL4 in AMs were analysed by RT-qPCR. **(F)** Western blot analysis of GPX4, SLC7A11, HO-1,and ACSL4 levels in AMs. The histogram showed the relative ratio of the expression levels of the target protein to that of an internal reference. Representative results from three independent experiments are shown (*n* = 3). Data are presented as the mean ± SEM. ns, no significance, *p < 0.05, **p < 0.01, ***p < 0.001,****p < 0.0001

Next, we aimed to elucidate the potential molecular mechanisms of plasma-EVs and hypothesized that activated plasma-EVs contribute to lung injury in sepsis. In vitro, we observed a significant increase in the expression of pro-inflammatory cytokines and the induction of M1 macrophage polarization in AMs co-cultured with plasma-derived LPS-induced ALI (LPS-EVs) (Fig. 4A). RT-qPCR analysis revealed an increase in AM autophagy in the LPS-EVs group (Fig. 4B), which was further confirmed by Western blot analysis (Fig. 4C). When these cells were co-cultured with LPS-EVs, the density of the mitochondrial membrane was significantly enhanced, and the mitochondrial volume was reduced, along with the fracture and loss of cristoids (Fig. 4D). Furthermore, the levels of GPX4 and SLC7A11 decreased while HO-1 and ACSL4 expression increased following the co-culture of LPS-EVs with AMs (Fig. 4E, F). Collectively, the above results suggested that LPS-EVs, containing pro-inflammatory EVs, induce autophagy and ferroptosis in AMs.

### LPS-EVs induce pulmonary inflammation, autophagy and ferroptosis in vivo

**Fig. 5:**
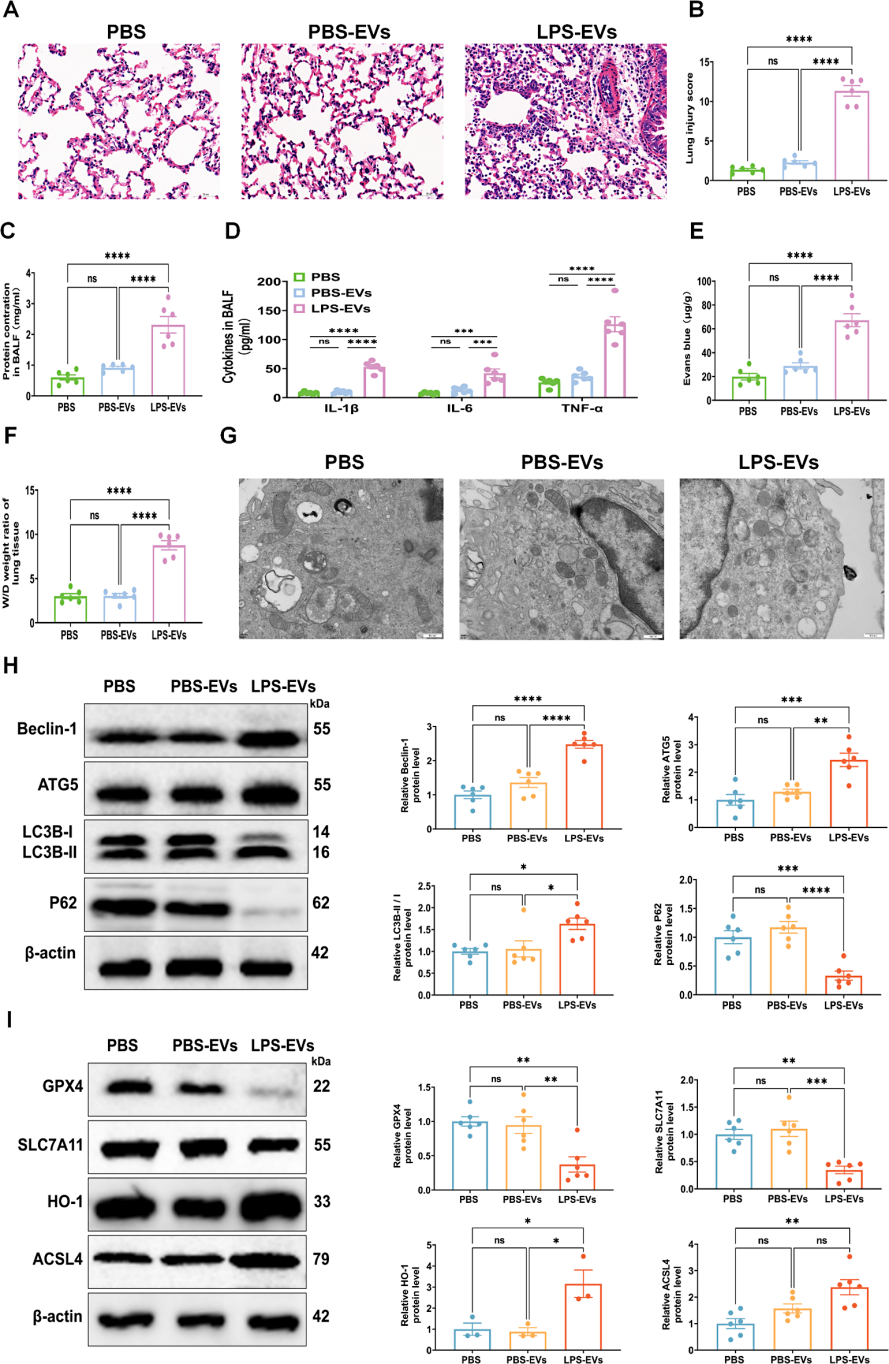
LPS-EVs induce lung injury by affecting AM autophagy and ferroptosis in vivo. **(A-B)** Representative H&E staining of lung tissues and the histogram showed the lung tissue pathological damage score. Scale bar, 50 μm. **(C)** The total protein concentration in BALF was detected by the BCA protein assay. **(D)** The concentrations of IL-1β, IL-6, and TNF-α in BALF were measured by ELISA. **(E)** Evans blue content in lung tissue. **(F)** The wet/dry weight ratio in lung tissue. **(G)** Mitochondrial damage and autophagosomes were detected by TEM. **(H)** The expressions levels of Beclin1, ATG5, LC3B-II/I, and p62 in lung were detected by Western blot. The histogram showed the relative ratio of the expression levels of the target protein to that of an internal reference. **(I)** The expression of GPX4, SLC7A11, HO-1, and ACSL4 in lung were detected by Western blot. The histogram showed the relative ratio of the expression levels of the target protein to that of an internal reference. Representative results from three independent experiments are shown (*n* = 6 rat/group). Data are presented as the mean ± SEM. ns, no significance, *p < 0.05, **p < 0.01, ***p < 0.001,****p < 0.0001

We conducted a study to investigate the potential involvement of activated LPS-EVs in the progression of ALI. We administered EVs obtained from the plasma of rats with LPS-EVs or PBS-EVs directly into their lungs for investigation. After 24 h, we harvested lung tissues from the rats. In histological analysis, the rats that were administered LPS-EVs displayed alveolar congestion, interstitial edema, and inflammatory cell infiltration in their lung tissues, whereas the rats that were given PBS or PBS-EVs exhibited minimal alterations (Fig. 5A, B). Moreover, there was an increase in total protein in the BALF of the rats (Fig. 5C). Compared with those in the PBS or PBS-EVs groups, the levels of TNF-α, IL-1β, and IL-6 in the BALF were significantly greater following LPS-EVs administration (Fig. 5D). An Evans blue dye assay revealed increased extravasation of dye from the lung vasculature into the interstitial and alveolar spaces in the LPS-EVs group (Fig. 5E). Similarly, the W/D ratio in the LPS-EVs group was significantly higher than that in the PBS and PBS-EVs groups (Fig. 5F). Additionally, LPS-EVs induced mitochondrial fragmentation, along with mitochondrial swelling and vacuolization, outer membrane rupture, and cristae disappearance (Fig. 5G). Furthermore, the expression of Beclin1, ATG5, and LC3B II/I was significantly upregulated, and that of P62 was downregulated in the lungs of the LPS-EVs group compared with those in the PBS or PBS-EVs groups (Fig. 5H). In addition, compared with those in the PBS or PBS-EVs group, the expression levels of GPX4 and SLC7A11 in the lung tissues of the LPS-EVs group were significantly lower, whereas those of HO-1 and ACSL4 were upregulated (Fig. 5I). Taken together, these findings demonstrate that LPS-EVs potentially induce pulmonary inflammation in vivo via AM autophagy and ferroptosis, which likely contributes to subsequent lung injury.

### Identification and validation of MEF2C as a downstream target of miR-223-3p

**Fig. 6:**
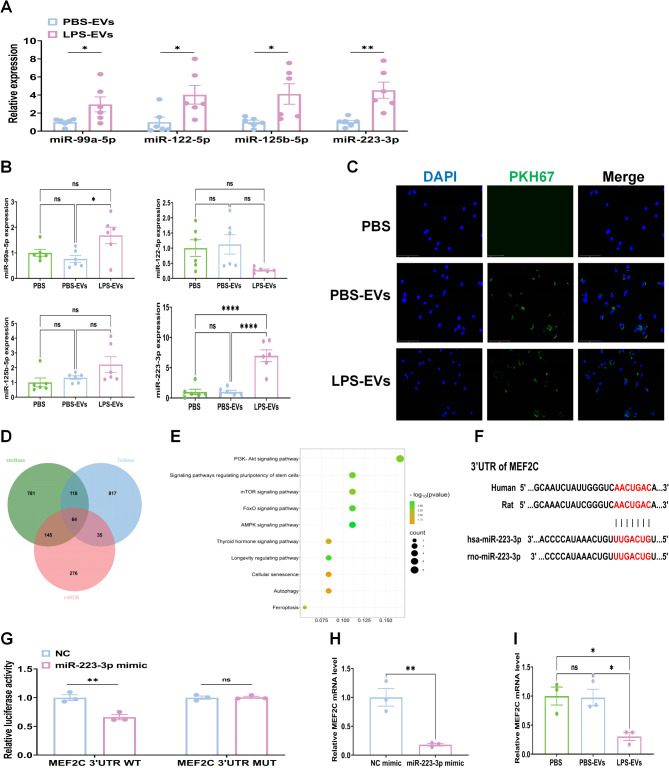
miR-223-3p activates AMs by targeting MEF2C. **(A)** RT-qPCR detection of the expression of the four selected miRNAs in plasma-EVs. **(B)** RT-qPCR detection of the expression of the four miRNAs in AMs co-cultured with PBS-EVs or LPS-EVs for 24 h. **(C)** Uptake of PKH67-labeled EVs (green) by AMs co-incubated for 6 h, observed under inverted fuorescence microscopy. **(D)** The intersection of predicted target genes of miR-223-3p from three databases (StarBase, TarBase, miRDB). **(E)** KEGG pathway analysis was performed on potential target genes of miR-223-3p. **(F)** Conservation of the miR-223-3p target sequence in MEF2C 3ʹUTR among different species and conservation of the miR-223-3p sequence among different species. **(G)** Dual-luciferase reporter assay performed in HEK293T cells. Cells were co-transfected with dual-luciferase reporter plasmids containing the wild-type or mutant MEF2C 3ʹUTR sequence, along with the NC or miR-223-3p mimic. **(H)** MEF2C expression was determined by RT-qPCR in cells transfected with miR-223-3p mimic or NC mimic 24 h post-transfection. **(I)** MEF2C expression was determined by RT-qPCR in AMs treated with PBS-EVs or LPS-EVs, respectively. Representative results from three independent experiments are shown (*n* = 6 except for *n* = 3 in G, H,I). Data are presented as the mean ± SEM. ns, no significance, *p < 0.05, **p < 0.01, ***p < 0.001,****p < 0.0001

In our preliminary studies, the EV miRNAs found in plasma samples from individuals with sepsis showed promising results, indicating that they warrant further investigation. We selected a group of sepsis-related miRNAs (miR-99a-5p, miR-122-5p, miR-125b-5p, and miR-223-3p) and analyzed their differential expression in rat plasma-EVs via RT-qPCR. The results demonstrated that miR-223-3p expression in LPS-EVs was the highest, revealing that plasma-EVs from septic rats selectively loaded with miR-223-3p (Fig. 6A). Interestingly, exposure to LPS-EVs led to the upregulation of miR-223-3p expression in AMs (Fig. 6B). These findings suggest that LPS can increase the loading of miR-223-3p into EVs from plasma and facilitate its transfer to recipient AMs. Using fluorescence microscopy, we noted the uptake of PKH67-labeled EVs by AMs (Fig. 6C). We used three databases (StarBase, TarBase, and miRDB) to predict potential target genes of miR-223-3p to clarify the impact and specific mechanism of exosomal miR-223-3p on AM autophagy and ferroptosis (Fig. 6D). KEGG pathway enrichment analysis revealed that the potential target genes of miR-223-3p were associated primarily with various pathways related to inflammation and cell death (Fig. 6E). Our hypothesis is that plasma-EVs could transfer miR-223-3p to lung tissues and then activate AMs during sepsis-induced ALI. Through bioinformatics analysis, it was found that the 3’UTR of MEF2C mRNA contains a conserved binding site for the seed region of miR-223-3p across different species (Fig. 6F). We propose that miR-223-3p targets MEF2C to downregulate its expression. In the dual-luciferase reporter assay, miR-223-3p significantly reduced the luciferase activity of the wild-type 3ʹUTR of MEF2C but had no inhibitory effect on the mutant 3ʹUTR of MEF2C (Fig. 6G). When the expression of MEF2C was compared between the two groups, a significantly lower level of expression was observed in AMs transfected with the miR-223-3p mimic than in those in the control group (Fig. 6H). More interestingly, mRNA detection revealed a significant decrease in MEF2C expression levels in AMs treated with LPS-EVs (Fig. 6I). Collectively, these findings suggest that miR-223-3p directly targets the 3ʹUTR of MEF2C mRNA, leading to the inhibition of MEF2C expression.

### mir-223-3p activates the Hippo signaling pathway by targeting MEF2C

**Fig. 7:**
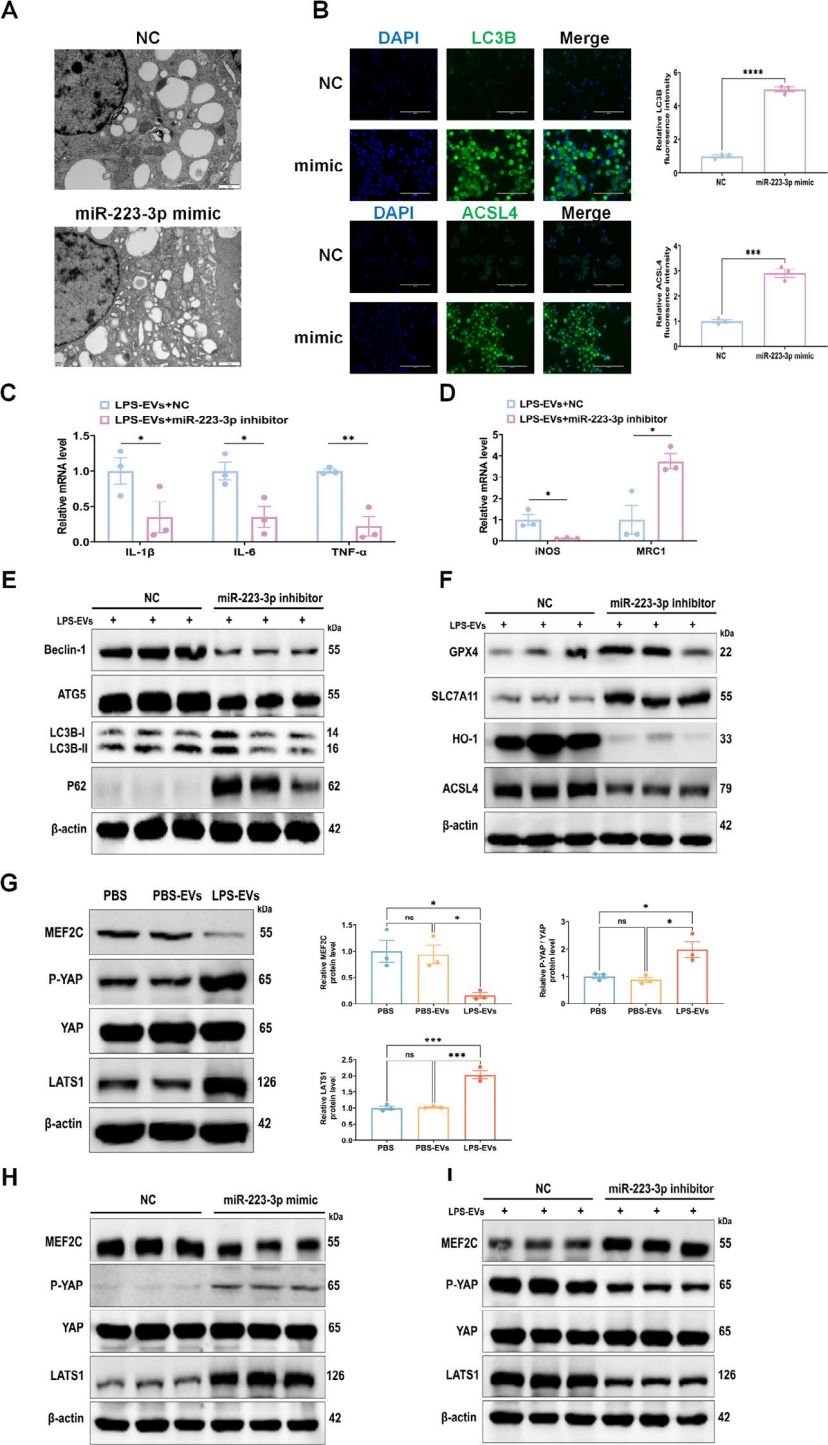
miR-223-3p in LPS-EVs promotes autophagy and ferroptosis in AMs via activating the Hippo signaling pathway in vitro. (**A**) Mitochondrial damage and autophagosomes were detected by TEM. (**B**) Representative images of LC3B and ACSL4, as observed by immunofluorescence assays, Scale bar, 1 μm. (**C**-**F**) Prior to co-culturing with LPS-EVs for 24 h, AMs were transfected with control or miR-223-p inhibitors for 24 h. c-d The mRNA expression levels of IL-1β, IL-6, TNF-α, iNOS and MRC1 in AMs were analysed by RT-qPCR. e Western blot analysis of Beclin-1, ATG5, LC3B-II/I, and P62 levels in AMs. f Western blot analysis of GPX4, SLC7A11, HO-1,and ACSL4 levels in AMs. (**G**) Treatment of AMs with PBS-EVs or LPS-EVs for 24 h. Western blot analysis of MEF2C, P-YAP/YAP, and LATS1 levels in AMs. The histogram showed the relative ratio of the expression levels of the target protein to that of an internal reference. (**H**) Western blot analysis of MEF2C, P-YAP/YAP, and LATS1 in AMs transfected with miR-223-3p mimics as indicated. (**I**) Prior to co-culturing with LPS-EVs for 24 h, AMs were transfected with control or miR-223-p inhibitors for 24 h. The expression levels of MEF2C, P-YAP/YAP, and LATS1 in AMs were measured by Western blot. Representative results from three independent experiments are shown (*n* = 3). Data are presented as the mean ± SEM. ns, no significance, *p < 0.05, **p < 0.01, ***p < 0.001,****p < 0.0001

A gain-or loss-of-function study was performed to validate miR-223-3p as a candidate for modulating MEF2C expression. Using transmission electron microscopy, we observed that compared with the NC group, the mitochondrial morphology of AMs in the miR-223-3p mimic group showed the characteristic changes of ferroptosis, including smaller mitochondria and reduced cristae (Fig. 7A). Immunofluorescence staining revealed an increase in autophagy and ferroptosis in AMs transfected with the miR-223-3p mimic (Fig. 7B). Additionally, a notable reduction in the expression of inflammatory markers and the activation of M1 macrophages was observed in AMs co-incubated with LPS-EVs and transfected with a miR-223-3p inhibitor (Fig. 7C, D). To assess the potential benefits of inhibiting miR-223-3p in lung injury, we investigated its impact on AM activity because of the crucial role of AMs in combating pathogens that cause septic lung injury in the clinic. When cocultured with LPS-EVs and transfected with a miR-223-3p inhibitor, the expression of Beclin-1, ATG5, and LC3BII/I was downregulated, whereas the expression of P62 was upregulated (Fig. 7E). Moreover, when cocultured with LPS-EVs and transfected with the miR-223-3p inhibitor, the expression of GPX4 and SLC7A11 was upregulated, whereas that of HO-1 and ACSL4 was downregulated (Fig. 7F). These results indicated that LPS-EVs from plasma promoted the autophagy and ferroptosis of AMs mediated by miR-223-3p. Furthermore, we observed a decrease in the expression of MEF2C and an increase in p-YAP and LATS1 in AMs coincubated with LPS-EVs compared with those in the control groups (Fig. 7G). Analysis of protein levels also revealed a decrease in MEF2C expression and an increase in p-YAP and LATS1 expression in AMs following transfection with the miR-223-3p mimic (Fig. 7H). After miR-223-3p inhibition, the effects of LPS-EVs on MEF2C, p-YAP, and LATS1 were reversed (Fig. 7I). These findings indicate that exosomal miR-223-3p can target and inhibit MEF2C expression, resulting in the activation of the Hippo signaling pathway, subsequently inducing autophagy and ferroptosis in AMs.

### mir-223-3p inhibition alleviates LPS-EV-induced lung injury

**Fig. 8:**
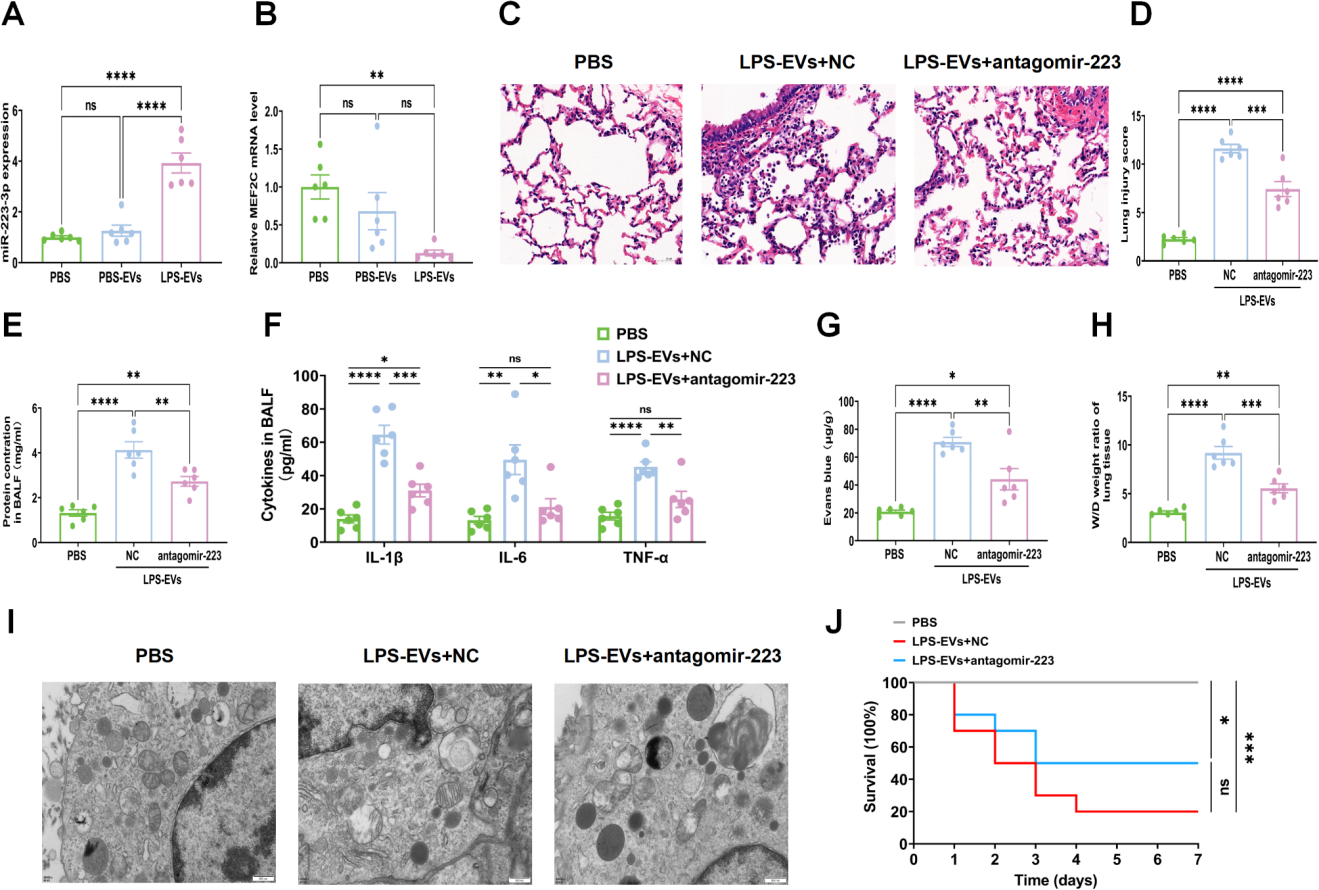
miR-223-3p inhibition alleviates LPS-EVs induced lung injury. The rats were injected intratracheally with LPS-EVs (300 µg/rat). These rats were sacrificed 24 h after LPS-EVs administration for the following experiments. **(A)** RT-qPCR detection of the expression of miR-223-3p in lung tissue. **(B)** RT-qPCR detection of the expression of MEF2C in lung tissue. **(C-D)** Representative H&E staining of lung tissues and the histogram showed the lung tissue pathological damage score. **(E)** The total protein concentration in BALF was detected by the BCA protein assay. **(F)** The concentrations of IL-1β, IL-6, and TNF-α in BALF were measured by ELISA. **(G)** Evans blue content in lung tissue. **(H)** The wet/dry weight ratio in lung tissue. **(I)** Mitochondrial damage and autophagosomes were detected by TEM. **(J)** Survival rate analysis of rats with or without miR-223-3p inhibition after LPS-EVs injection (*n* = 10), was performed using the log-rank test. Data are presented as the mean ± SEM. ns, no significance, *p < 0.05, **p < 0.01, ***p < 0.001,****p < 0.0001

Next, we further investigated the functional role of miR-223-3p in the pathogenesis of lung injury. After the administration of LPS-EVs, we observed a significant increase in miR-223-3p expression in the lungs compared with that in the lungs of those treated with PBS-EVs (Fig. 8A). In addition, mRNA analysis demonstrated a notable reduction in MEF2C expression levels in the lungs following LPS-EV treatment (Fig. 8B). Prior to the injection of LPS-EVs, the rats were transfected with antagomiR-223-3p (a miR-223-3p inhibitor) or NC inhibitor, after which inflammation and injury in the lung tissues were assessed. Histological analysis revealed that transfection with antagomiR-223-3p reduced inflammatory cell infiltration, thickening of the alveolar septa, and exudation (Fig. 8C, D). There was a decrease in total protein in the BALF of the rats (Fig. 8E). The expression of the pro-inflammatory cytokines IL-1β, TNF-α, and IL-6 also decreased in the BALF (Fig. 8F). Moreover, the extravasation of Evans blue dye from the lung vasculature into the interstitial and alveolar spaces was lower in the LPS-EV + antagomiR-223-3p group than in the LPS-EV + NC group (Fig. 8G). The W/D ratio of the LPS-EVs + antagomiR-223-3p group was significantly lower than that of the LPS-EVs + NC group (Fig. 8H). Strikingly, the mitochondria observed via transmission electron microscopy had shrunk in size, the cristae had decreased or disappeared, and the outer membrane had ruptured in the LPS-EVs + NC group, but LPS-EV + antagomiR-223-3p alleviated ferroptosis (Fig. 8I). After LPS-EV stimulation, we also observed a trend toward longer survival times in rats treated with antagomiR-223-3p than in those without inhibition, although this difference did not reach statistical significance (Fig. 8J). Together, the suppression of miR-223-3p may mitigate LPS-EV-induced lung injury, diminish pulmonary inflammation, and prevent macrophage death.

## Discussion

In the present study, several significant findings were revealed. First, we mapped the miRNA and protein expression profiles of EVs derived from septic plasma. Our findings revealed that miRNAs and PMN-related proteins from plasma-EVs could serve as potential diagnostic and prognostic biomarkers for septic ARDS. Second, EVs derived from plasma in LPS-induced ALI, which contain pro-inflammatory EVs, facilitate inflammation and the death of AMs. Additionally, we found that miR-223-3p is upregulated in LPS-EVs, leading to the activation of Hippo signaling and triggering autophagy and ferroptosis in AMs via the targeting of MEF2C. Finally, to further validate the role of miR-223-3p, we injected a miR-223-3p antagomir into rats to verify the protective effect of inhibiting miR-223-3p on AMs and its ability to reverse LPS-EV-induced lung injury in vivo. These results suggest that LPS-EVs play crucial pathogenic roles in mediating pulmonary inflammation and tissue injury, indicating their involvement in the progression of septic ALI.

EVs are nanoscale particles released by cells to facilitate intercellular communication, making them an attractive means for diagnosing disease, predicting its progression, and evaluating its response to treatment (Das et al. 2024). Researchers have shown that circulating exosomal miRNAs can serve as early predictors of persistent organ failure in acute pancreatitis (Li et al. 2024). Circulating exosome-derived miR-155 promotes macrophage proliferation and inflammation (Jiang et al. 2019); however, the role of exosomal proteins, such as PMN-related proteins in sepsis-induced ALI, remains unclear. Neutrophils are essential in the innate immune response. Olfactomedin 4 (OLFM4) is produced by a specific group of neutrophils, and increased OLFM4 expression has been linked to worse results in individuals with sepsis and ARDS, influencing the body’s inflammatory reaction after tissue damage (Kangelaris et al. 2015; Kassam et al. 2021). Previous studies have reported that OLFM4 may modulate metabolic disorders to regulate the pro-inflammatory response of lung epithelial cells in sepsis-induced ALI (Gong et al. 2021). Additionally, Lipocalin-2 (LCN2) belongs to the lipocalin family and acts as a primary controller of iron metabolism, oxidative stress, and inflammation in mammals (Kang et al. 2018; Xiao et al. 2017). Suppression of LCN2 reduces ARDS by disrupting ferroptosis-induced lung inflammation and oxidative stress through the inhibition of MAPK/ERK signaling (Wang et al. 2022). Moreover, hepatic ischemia-reperfusion (HIR)-Exos deliver liver-specific miR-122-5p to AMs, resulting in the induction of ALI by promoting M1 macrophage polarization (Lyu et al. 2024). Our results suggest that miRNAs and PMN-related proteins from plasma-EVs could potentially serve as diagnostic and prognostic biomarkers for septic ARDS, but whether miR-122-5p, OLFM4, and LCN2 are involved in the pathogenic mechanism of septic ARDS has yet to be explored.

AMs play a crucial role as the primary innate immune cells in the respiratory system, contributing to the protection of the host against pathogens and maintaining tissue balance (Aegerter et al. 2022). Research has demonstrated that EVs derived from the plasma of mice with acute pancreatitis can induce NLRP3-dependent pyroptosis in AMs (Wu et al. 2020). When EVs containing phagocytosed MRSA are released, AMs demonstrate notable proinflammatory impacts and trigger necroptosis through the transmission of TNF-a and miR-146a-5p (Bai et al. 2024). Similarly, miR-155-5p in EVs promotes widespread M1 polarization of macrophages in hypervirulent *Klebsiella* pneumoniae-induced ALI through the MSK1/p38-MAPK axis (Xu et al. 2023). In contrast, M2-like macrophage-derived EVs play a protective role in the pathogenesis of ALI/ARDS, which is partly mediated by miR-709 (Yang et al. 2024). Therefore, targeted macrophage polarization in this context can effectively regulate the secretion of inflammatory factors, thus leading to a reduction in tissue damage and patient mortality. In the present study, AMs were the key effector cells through which LPS-EVs mediate autophagy and ferroptosis, suggesting their potential as a target for therapeutic approaches to mitigate the lung pathologies associated with sepsis.

Autophagy is triggered by reactive oxygen species (ROS) during ferroptosis, which results from an overabundance of iron or the buildup of lipid-dependent ROS. This mechanism subsequently enhances the accumulation of iron and ROS, establishing a cycle that contributes to the hastening of ferroptosis (Lee et al. 2023). The correlation between autophagy and ferroptosis has garnered increasing interest, offering a new perspective on the regulation of cell death. Mounting evidence indicates that autophagy may contribute to ferroptotic cell death, particularly under specific conditions (Liu et al. 2020). These findings suggest that autophagy may increase the production of ROS during ferroptosis by regulating key pathways related to iron metabolism and lipid peroxidation (Yang et al. 2023; Sun et al. 2021; Hou et al. 2016). These reports indicate that ferroptosis may be a form of autophagic cell death in certain circumstances. Current evidence suggests that autophagy can exert both promotive and protective effects on ferroptotic cell death (Chen et al. 2024), yet the underlying mechanisms and checkpoints regulating septic ARDS remain poorly elucidated. By elucidating the complex interaction between autophagy and ferroptosis, we can lay the groundwork for precise sepsis therapies.

The findings of the in vivo experiments revealed similar trends in clinical patients with septic ARDS. Notably, the level of miR-223-3p is significantly elevated in plasma-EVs from patients with septic ARDS. The role of miR-223-3p in disease is still a matter of debate. In mouse models of LPS-induced ALI, miR-223-3p carried by BALF-exos reduces ALI by targeting STK39 in AMs (He et al. 2022). EVs derived from mesenchymal stromal cells can reduce the inflammation of LPS-induced lung epithelial cells by delivering miR-223-3p to the cells (Chen et al. 2023). However, it has been suggested that miRNA-223 could contribute to the pathogenicity of influenza virus, as a notable increase in its expression levels was observed in the lungs of mice infected with either the recombinant 1918 pandemic virus or a highly virulent strain (Li et al. 2010). Additionally, blocking miRNA-223 led to a decrease in the death rate of infected mice (Choi et al. 2014). These results indicate that a specific miRNA could exhibit varying functions as a result of targeting different genes. During the course of infection, miRNA-223 is involved in restricting the activation of the NLRP3 inflammasome and cytokine-driven inflammation (Fang et al. 2006; Roux et al. 2013). Notably, miR-223-deficient mice presented increased levels of the NLRP3 protein in vivo (Baek et al. 2008), which correlated with neutrophilia, inflammatory lung disease (Johnnidis et al. 2008), and increased CXCL2 levels (Dorhoi et al. 2013). Emerging evidence indicates that plasma-EVs can also trigger inflammatory responses, leading to ALI after being taken up by AMs (Wu et al. 2020). Recent studies have demonstrated that host EVs in the bloodstream can capture LPS, leading to non-canonical inflammasome activation and pyroptosis(Kumari et al. 2023). It remains unclear whether EV-facilitated cytosolic access of LPS contributes to the poor prognosis and high mortality of septic ARDS, and further studies are warranted in the future. In the present study, upregulated miR-223-3p promoted inflammation and cell death in rats with LPS-EV-induced lung injury. Therefore, a miR-223-3p antagomir was used to treat lung injury. Functionally, inhibition therapy targeting miR-223-3p further mitigated pulmonary inflammation and cell death in rats with LPS-EV-induced lung injury. Together, these findings indicate that specifically inhibiting miR-223-3p could be a potentially effective intervention for treating sepsis-induced ALI, providing novel opportunities to alleviate lung damage in patients with sepsis.

However, how the upregulation of miR-223-3p in LPS-EVs promotes autophagy and ferroptosis is unknown. Prior studies have shown that MEF2 is linked to inflammatory disorders, particularly emphasizing that reducing MEF2C can increase the production of pro-inflammatory cytokines and chemokines (Deczkowska et al. 2017). In contrast, increased MEF2C levels hinder the activation of pro-inflammatory molecules (Xu et al. 2015). Recent research has revealed a reduction in MEF2C expression in rats with CLP-induced sepsis, and increasing MEF2C levels can mitigate ALI, reduce lung inflammation, and reduce apoptosis (Liang et al. 2021). Moreover, the Hippo-YAP pathway plays a crucial role in regulating organ size, tissue homeostasis, and regeneration, with significant biological implications (Piccolo et al. 2014). The elements of the Hippo-YAP pathway, including MST1/2, NDR1/2, and YAP/TAZ, are essential regulators of innate immunity (Wang et al. 2020). More notably, MST1/2 can detect ROS and safeguard macrophages from oxidative stress by regulating the stability of Nrf2, an antioxidant transcription factor. Deficiencies in MST1/2 lead to increased ubiquitination of Nrf2 and reduced expression of antioxidant genes, leading to increased oxidative stress, aging, and death of phagocytes (Wang et al. 2019). Generally, recent research has suggested that there is mutual activation between ROS and Hippo-YAP signaling. In line with the findings of the aforementioned study, our research revealed that upregulated miR-223-3p in EVs can increase Hippo signaling by targeting MEF2C, thereby promoting LPS-EV-induced autophagy and ferroptosis in AMs.

Our study had several limitations. First, in the proteomic analysis of EVs, our focus was solely on their clinical significance in terms of differential expression, without further validation of the potential role of other differentially expressed proteins in regulating inflammation and cell death. Second, since our model of ALI was established by intratracheally injecting LPS to obtain LPS-derived plasma EVs, it might be more conducive to observe the effect of EVs on alveolar macrophages directly by intratracheal injection of EVs as well, but this may deviate from the typical pathway of EV release and uptake in lung tissue. A more comprehensive comparison of different delivery modes of EVs warrants further investigation in future studies. Third, in our miRNA analysis, we specifically focused on miR-223-3p because of its relatively high basal expression level, but it is important to consider other differentially expressed miRNAs such as miR-99a-5p. Hence, a thorough examination of the distinctively expressed proteins and miRNAs in EVs is essential for further investigations into the pathogenesis of septic ALI. Finally, the controversy surrounding the role of miR-223-3p in ALI may stem from variations in ALI models and observation times, prompting an intriguing question that warrants further investigation in the future.

## Conclusions

**Fig. 9:**
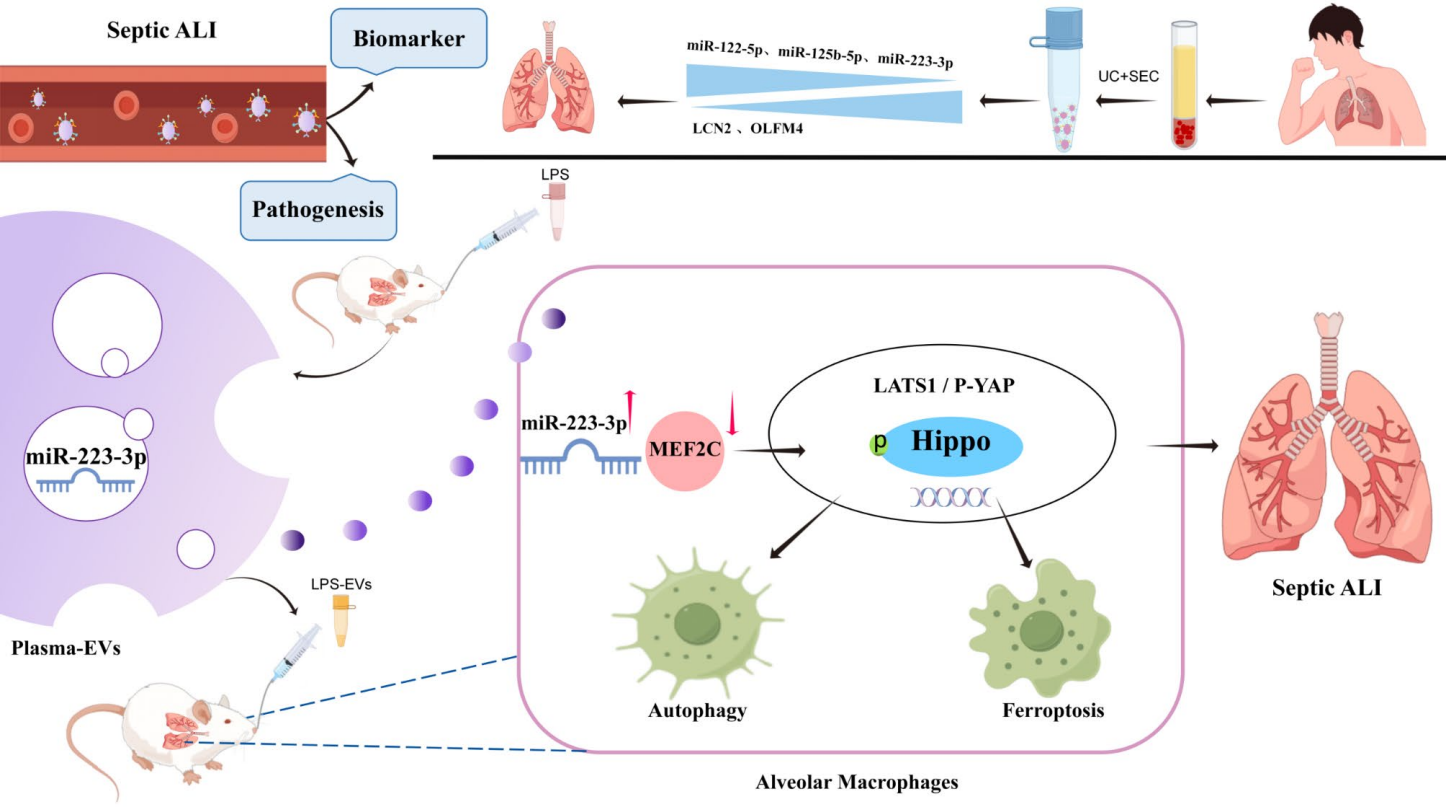
Flow chart. The differential expression analysis of miRNAs and PMN-related proteins indicated that the plasma-EV model has potential for predicting septic ARDS and prognosis among septic patients. Schematic representation of the mechanism by which plasma-derived EVs induce AM autophagy and ferroptosis in septic ALI. Created with Figdraw.com

Our findings provide evidence that miR-223-3p derived from the plasma-EVs of LPS-induced ALI triggers autophagy and ferroptosis in AMs by activating the Hippo signaling via targeting MEF2C. Overall, this study enhances the understanding of the mechanisms underlying septic ALI, provides clues for modulating the cross-talk between plasma and AMs, and highlights a plausible strategy for assessing septic progression and treating lung injury (Fig. 9).

## Electronic supplementary material

Below is the link to the electronic supplementary material.

Supplementary Material 1

Supplementary Material 2

## Data Availability

No datasets were generated or analysed during the current study.

## Abbreviations

AMs: Alveolar Macrophages
ALI: Acute Lung Injury
ARDS: Acute Respiratory Distress Syndrome
APACHE: Acute Physiology And Chronic Health Evaluation
CLP: Cecal Ligation And Puncture
EVs: Extracellular Vesicles
ELISA: Enzyme-Linked Immunosorbent Assay
GO: Gene Ontology
HIR: Hepatic Ischemia-Reperfusion
H&E: Hematoxylin and Eosin
IL: Interleukin
KEGG: Kyoto Encyclopedia of Genes and Genomes
LPS: Lipopolysaccharide
LCN2: Lipocalin-2
miRNA: microRNA
MEF2C: Myocyte Enhancer Factor 2 C
NanoFCM: Nano Flow Cytometry
OLFM4: Olfactomedin 4
PMNs: Polymorphonuclear Neutrophils
PBS: Phosphate-Buffered Saline
ROS: Reactive Oxygen Species
ROC: Receiver Operating Characteristic Curve
RT-qPCR: Real-Time quantitative Polymerase Chain Reaction
SEC: Size Exclusion Chromatography
SOFA: Sequential Organ Failure Assessment
SLPI: Secretory Leukoprotease Inhibitor
TEM: Transmission Electron Microscopy
UC: Ultracentrifugation
W/D: Wet-to-Dry weight
