# Supplementary figures and images for: Plasma-derived extracellular vesicles prime alveolar macrophages for autophagy and ferroptosis in sepsis-induced acute lung injury
Source: Mol Med. 2025 Feb 4;31:40. doi: 10.1186/s10020-025-01111-x (PMC11792199; doi:10.1186/s10020-025-01111-x)

Fig 1F

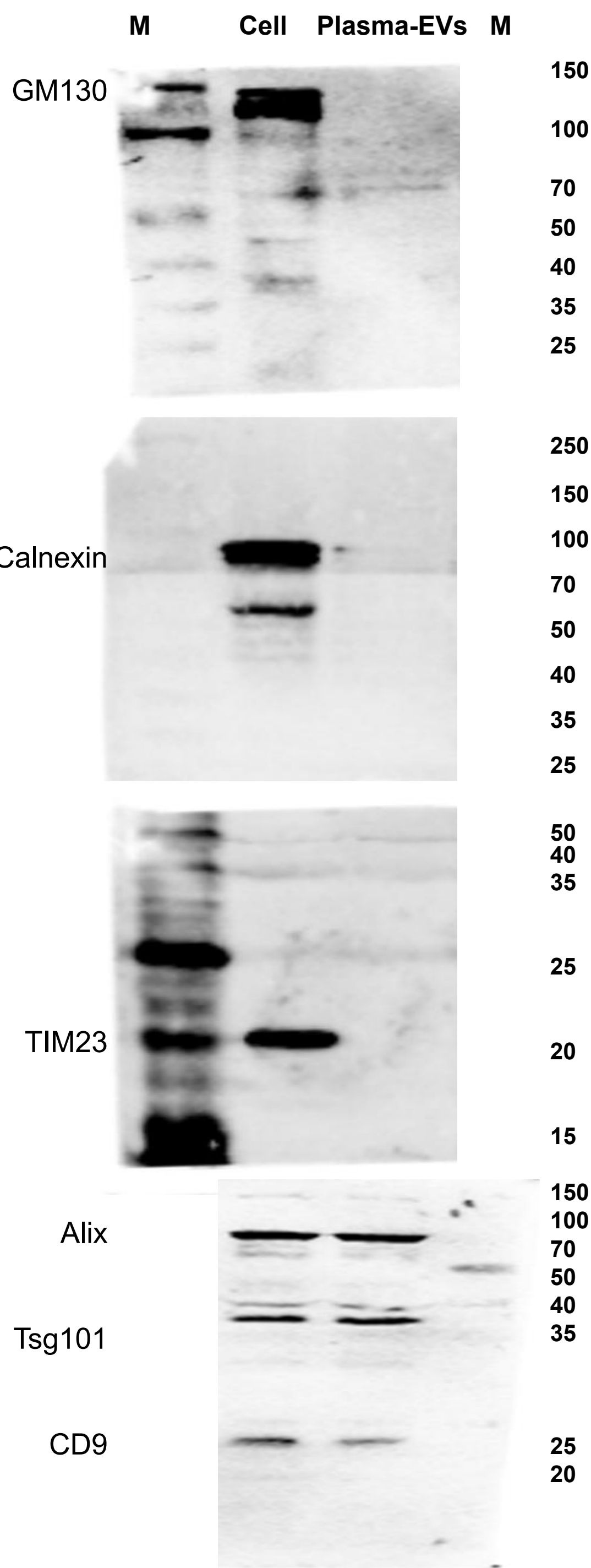

Fig 4C

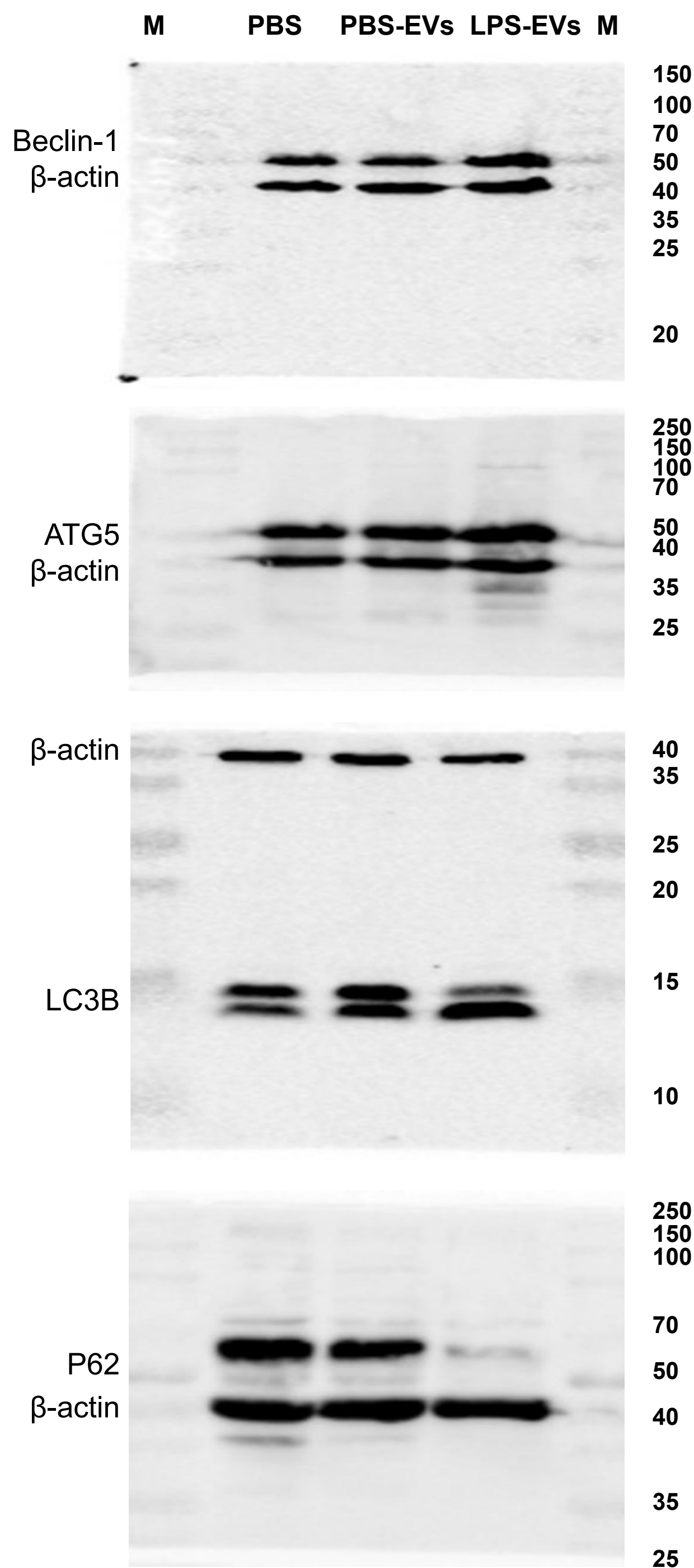

Fig 4F

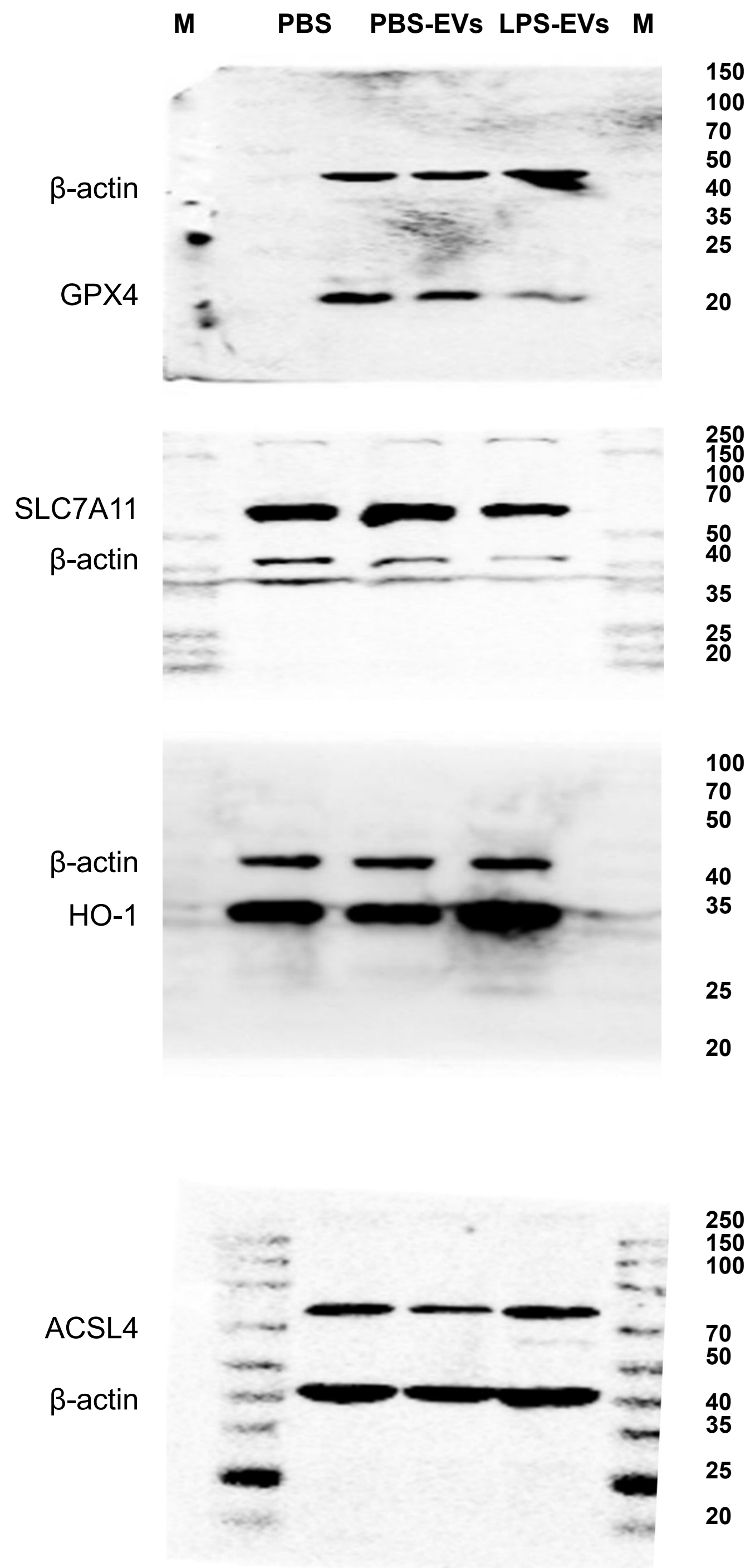

Fig 5H

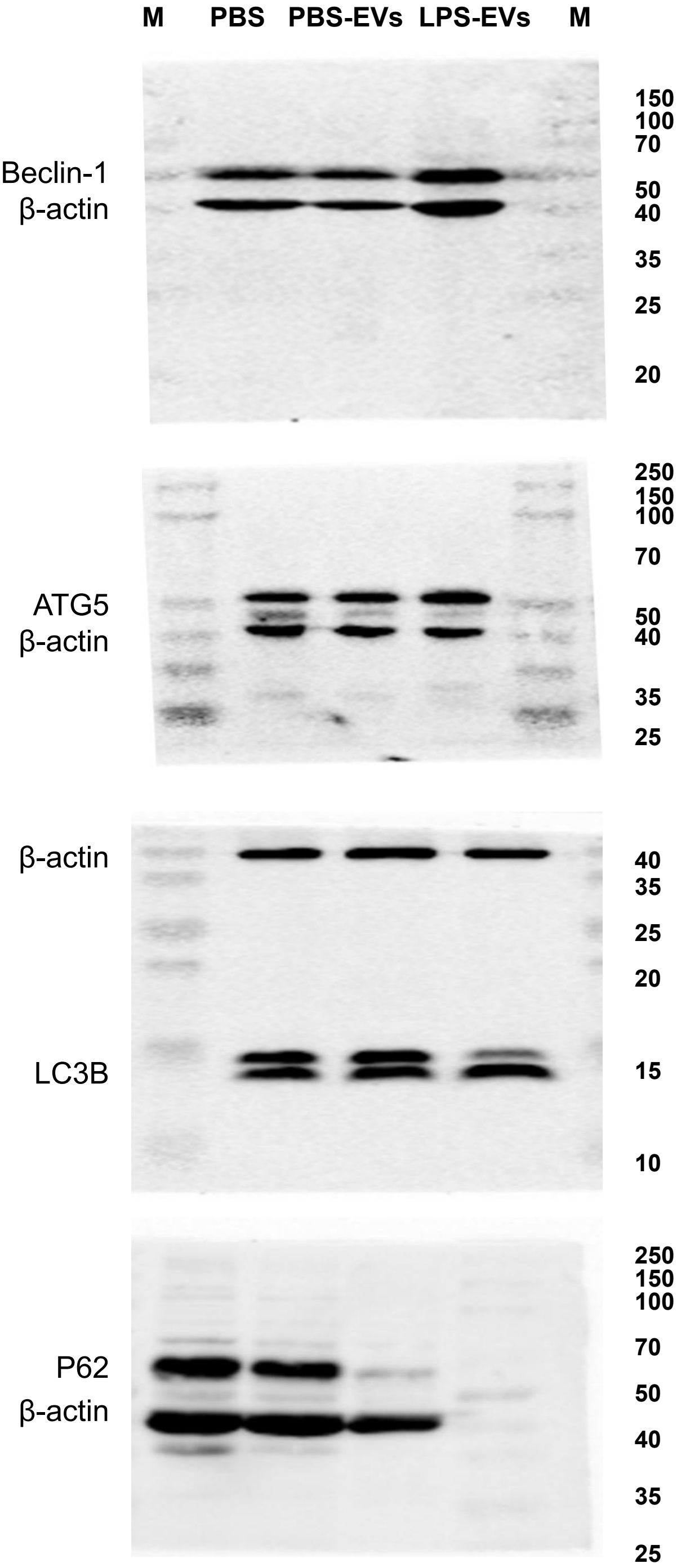

Fig 5I

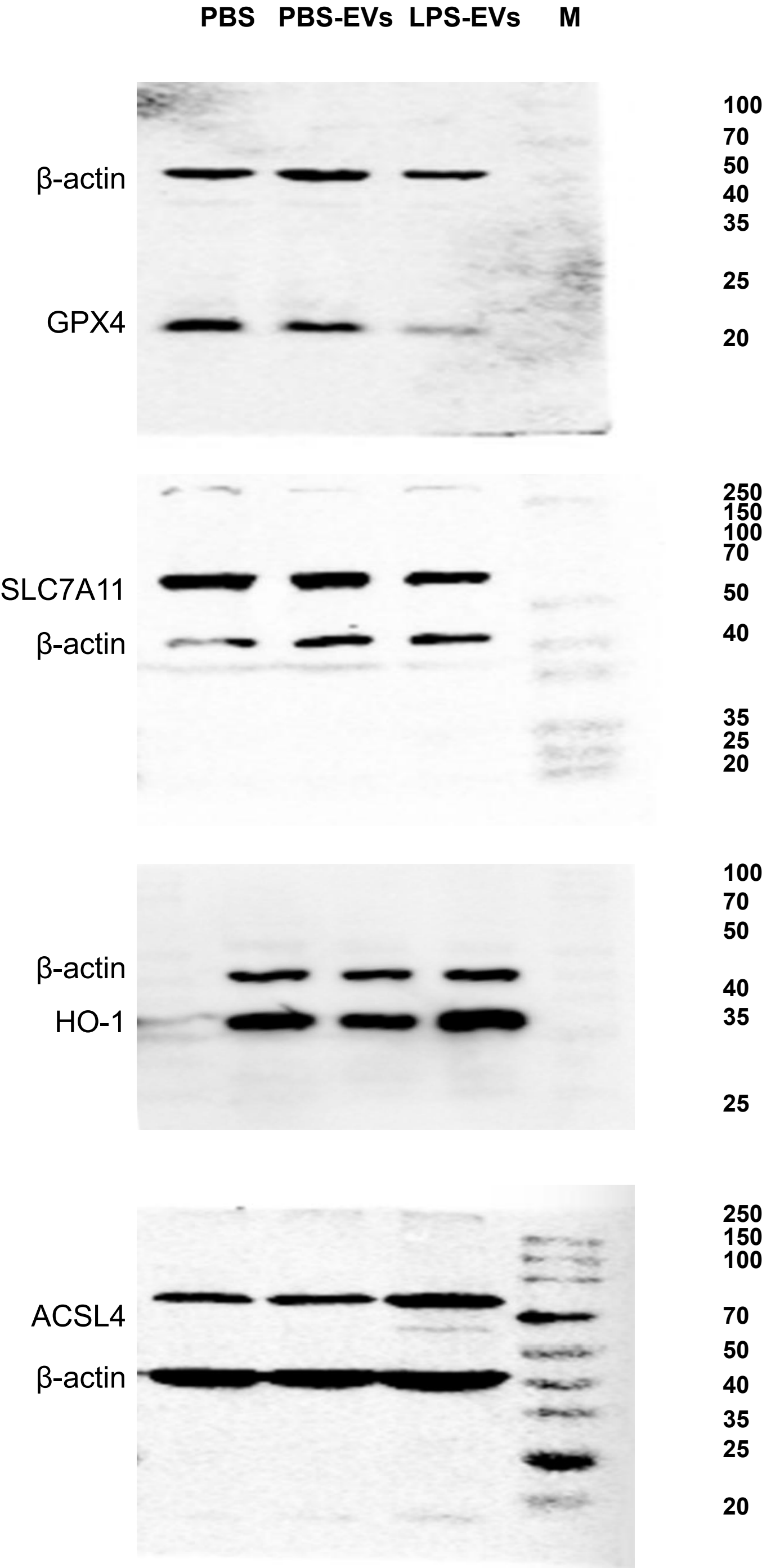

Fig 7E

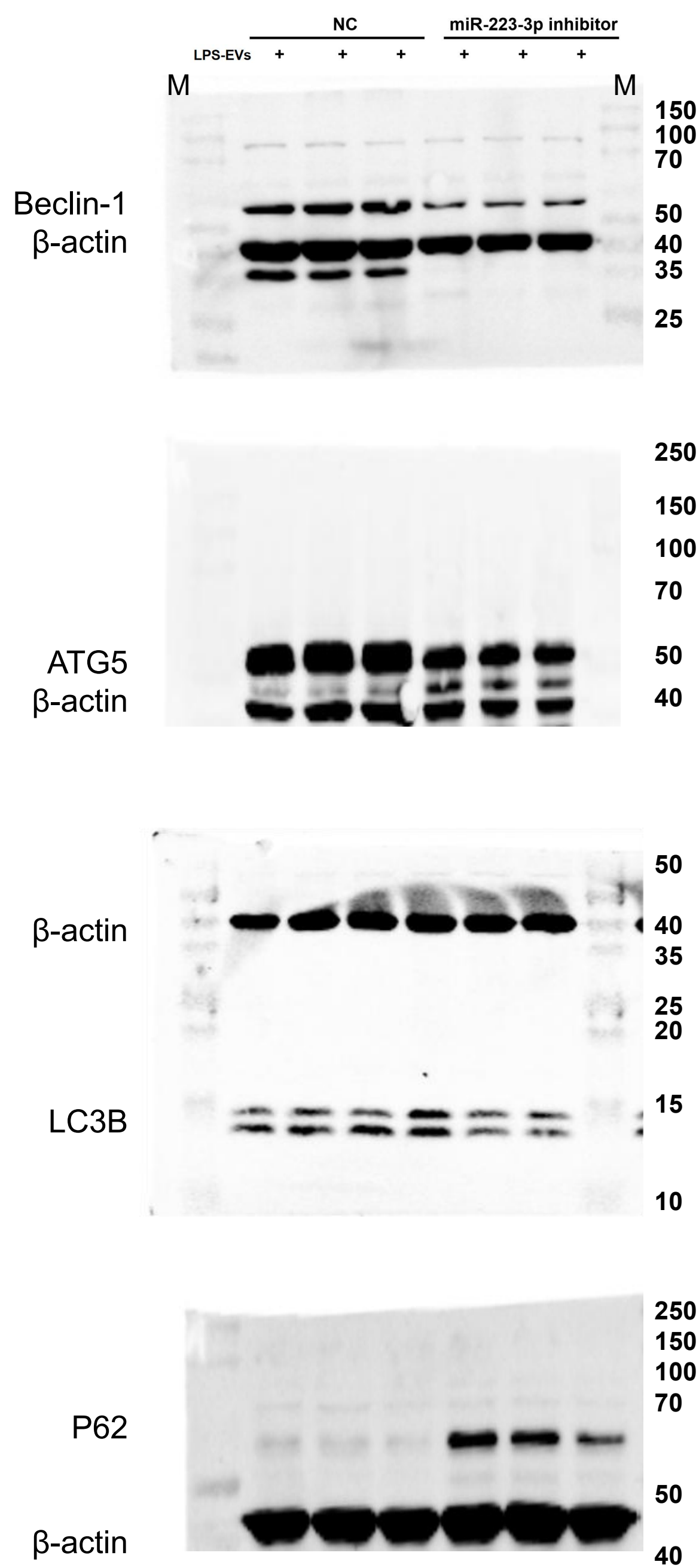

Fig 7F

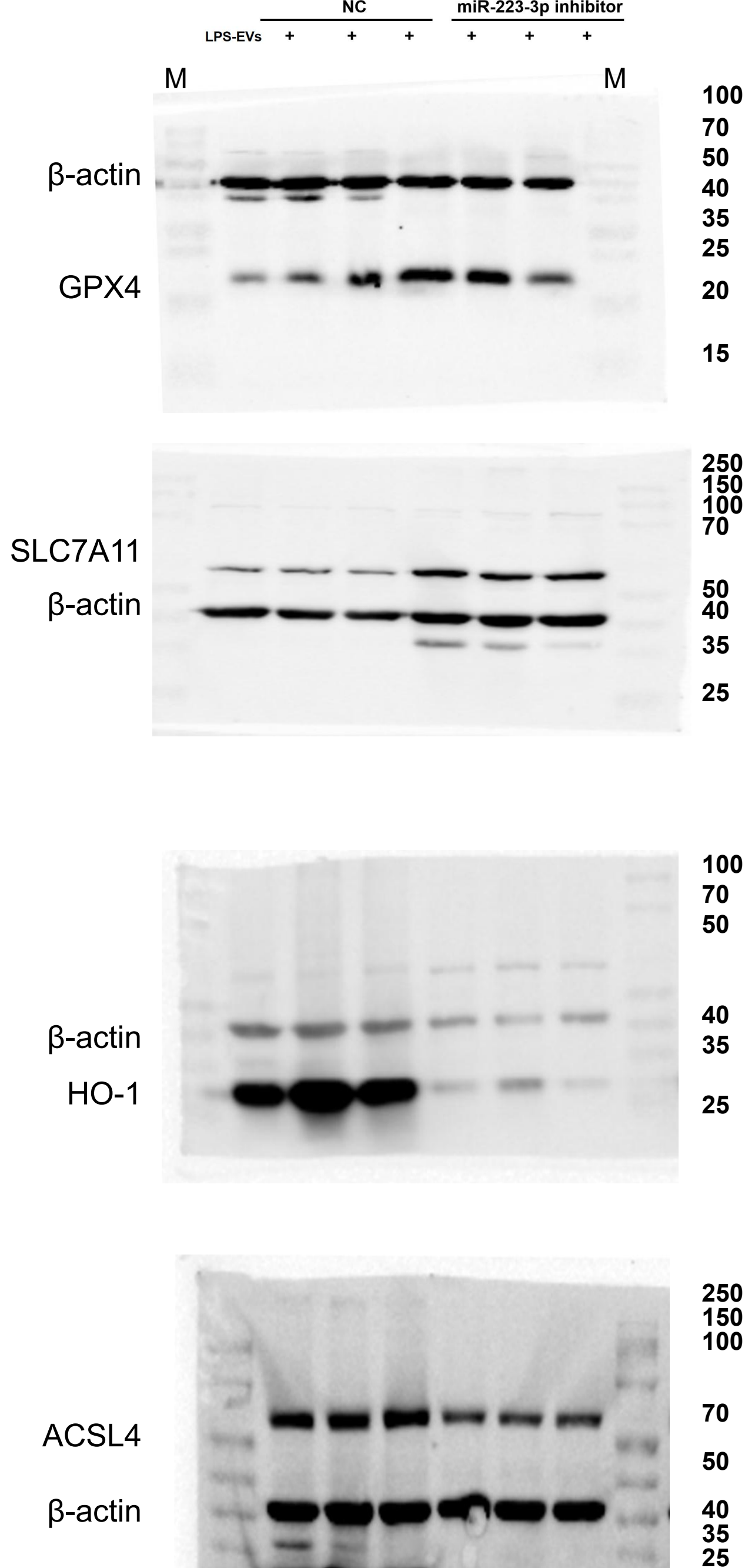

Fig 7G

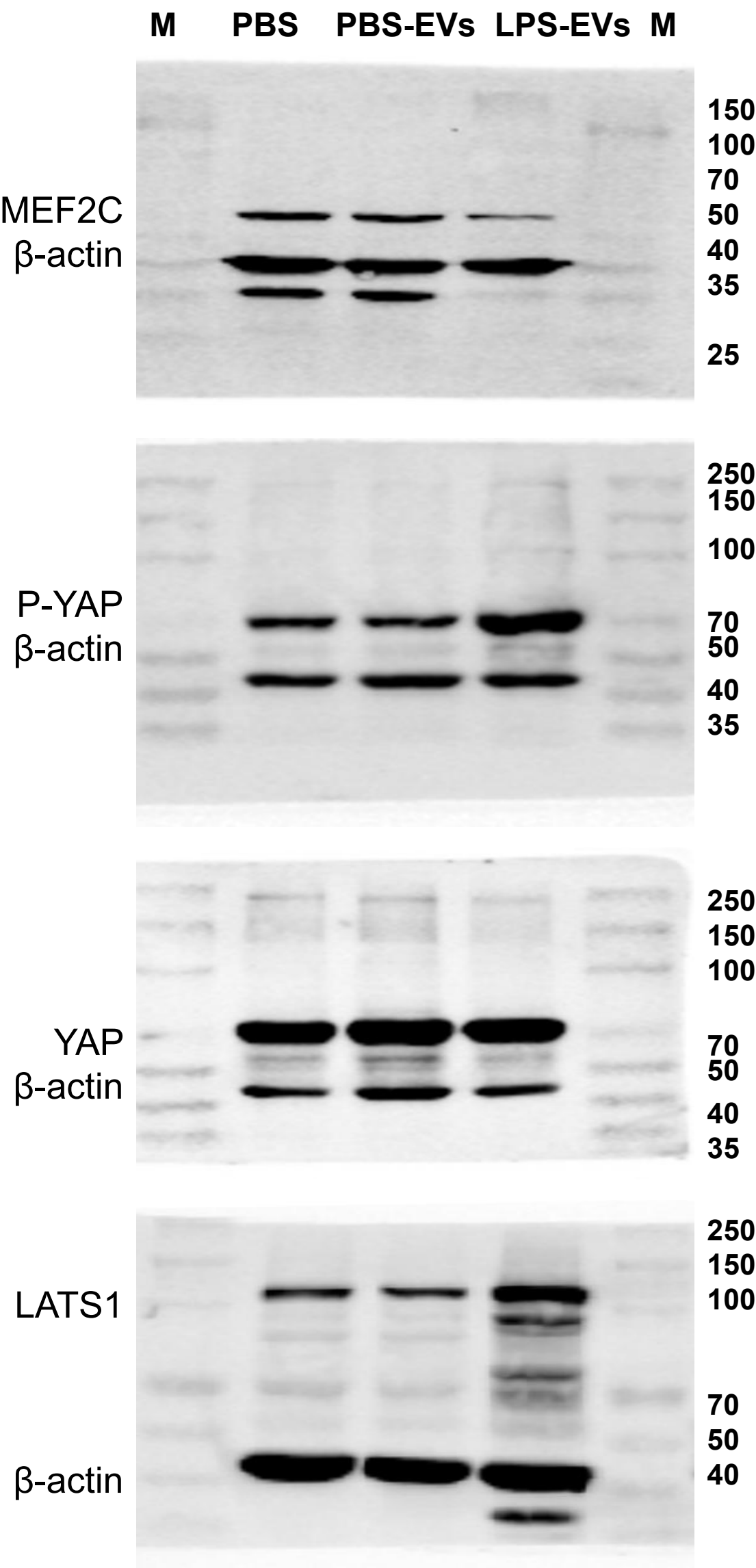

**Fig 7H**

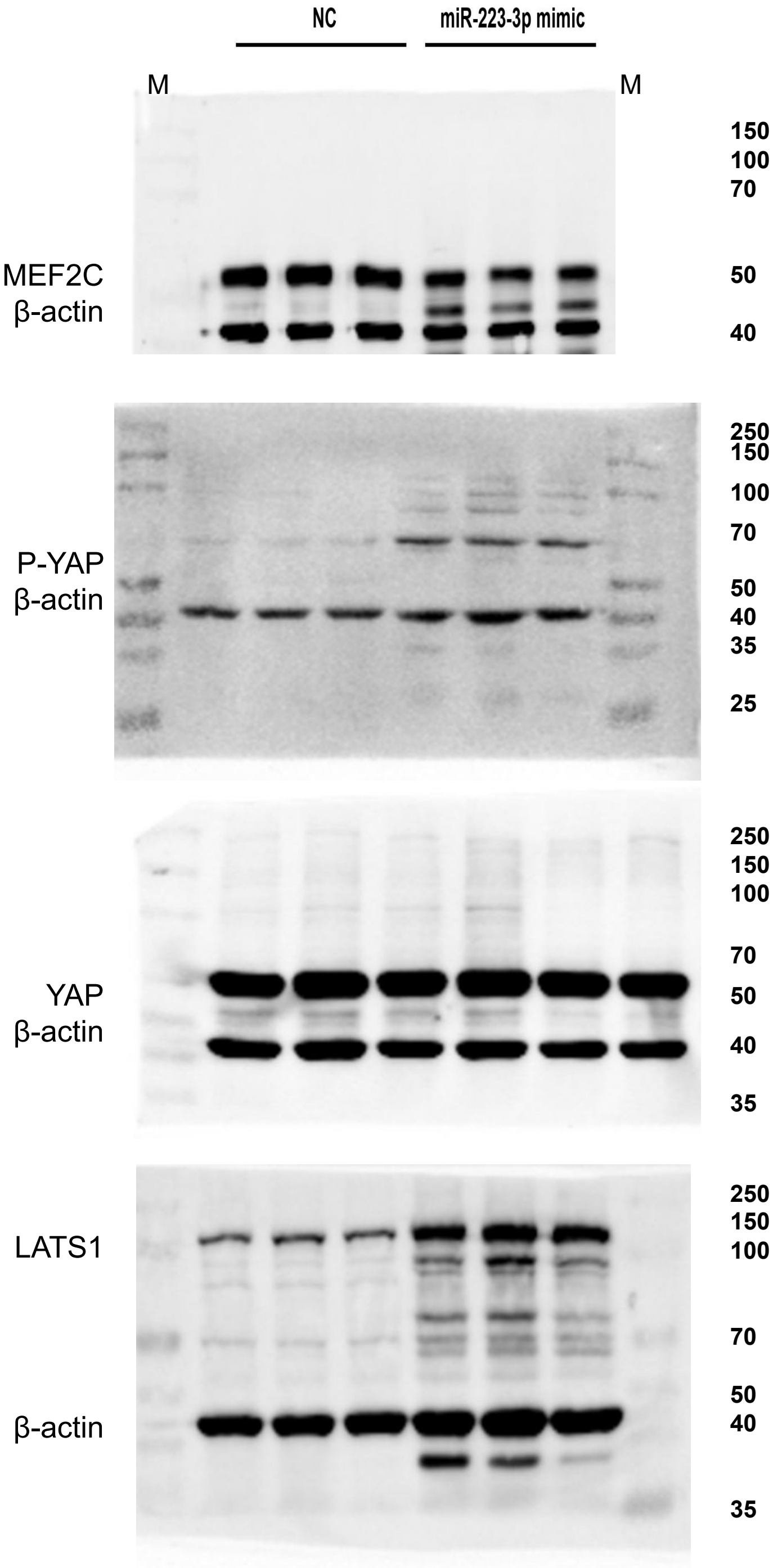

**Fig 7I**

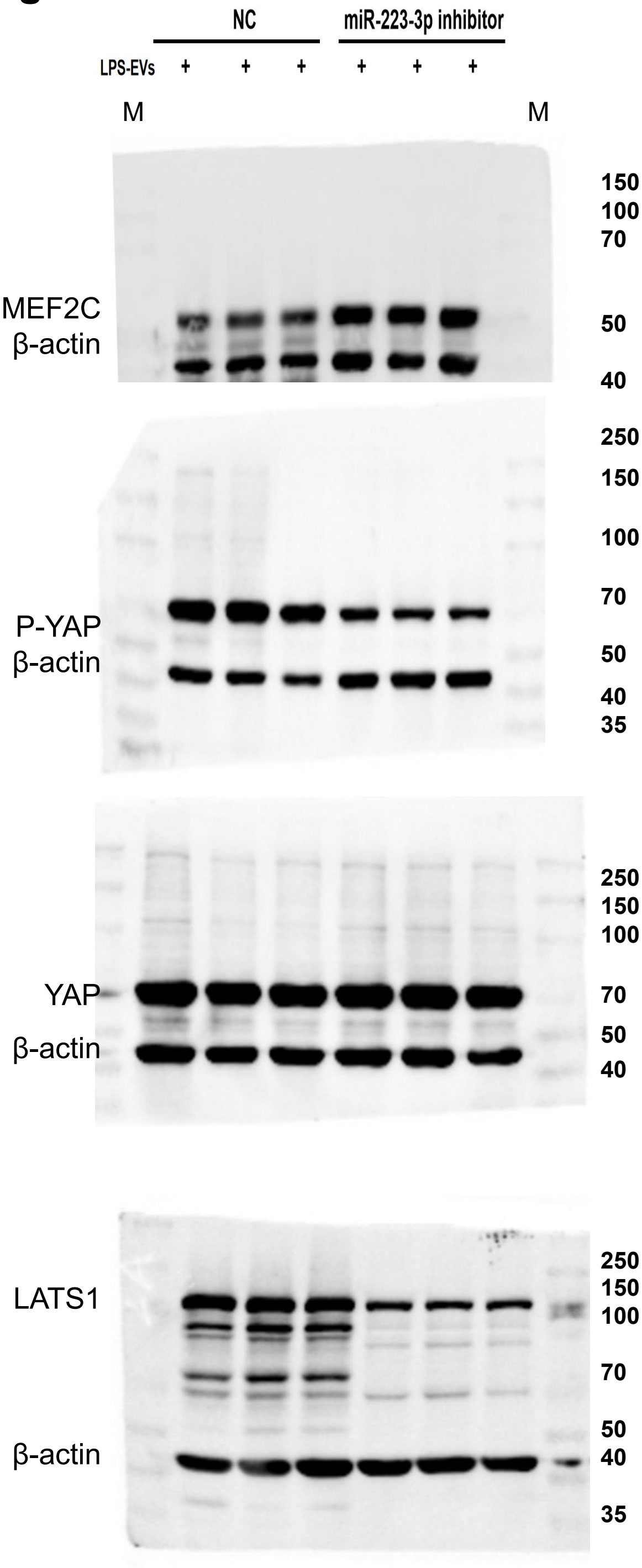

Fig S4I

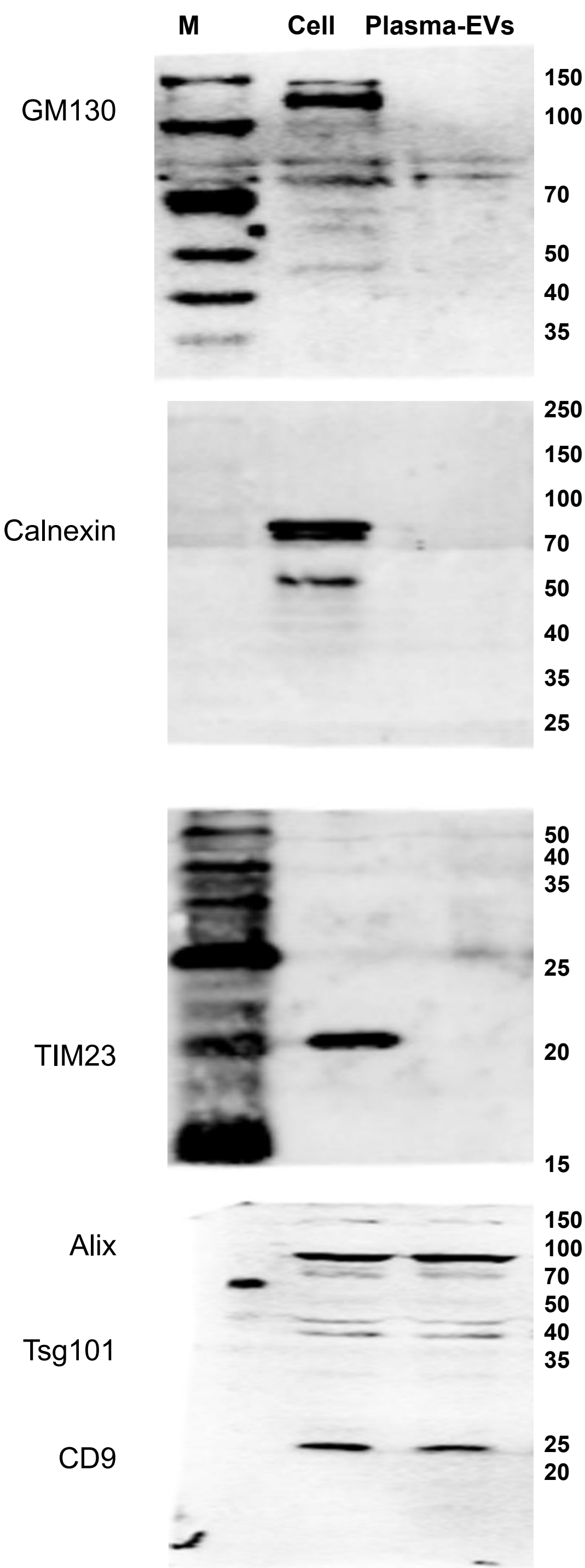

Supplement: Supplementary file 1 — Supplementary Material 1 [file 10020_2025_1111_MOESM1_ESM.pdf]
