# Supplementary material for: Plasma-derived extracellular vesicles prime alveolar macrophages for autophagy and ferroptosis in sepsis-induced acute lung injury
Source: Mol Med. 2025 Feb 4;31:40. doi: 10.1186/s10020-025-01111-x (PMC11792199; doi:10.1186/s10020-025-01111-x)
Supplement: Supplementary file 2 — Supplementary Material 2 [file 10020_2025_1111_MOESM2_ESM.docx]

**Fig.S1 Validation of miRNA and PMN-related protein expression in plasma-EVs from sepsis patients, septic shock patients and healthy controls.** **(A)** Validation of the expression levels of miR-30e-3p, miR-99a-5p, miR-122-5p, miR-125b-5p, miR-150-5p, miR-155-5p, miR-223-3p, and miR-378a-3p in plasma-EVs by RT-qPCR. **(B)** Validation of the expression levels of PMN-related proteins (CD177, SLPI, OLFM4 and LCN2) in plasma-EVs by ELISA. Data are presented as the mean ± SEM. ns, no significance, **p* < 0.05, ***p* < 0.01, ****p* < 0.001,*****p* < 0.0001.

**Fig.S2 Spearman correlation analysis.** Spearman correlation between the levels of miR-122-5p, miR-125b-5p, miR-223-3p, LCN2, and OLFM4 in plasma-EVs of septic patients and their **(A)** SOFA score, **(B)** APACHE II score, **(C)** PaO_2_/FiO_2_, and **(D)** duration of mechanical ventilation.

**Fig.S3 Diagram of the regulatory network of TF-miRNA-mRNA target interactions. (A)** The intersection of the predicted target genes of miR-122-5p, miR-125b-5p, and miR-223-3p from three databases (TargetScan, miRDB, and miRTarBase). The 3 upregulated miRNAs were predicted to target TFs from the TRRUST database. TF-miRNA-mRNA regulatory networks were constructed for sepsis on the basis of the above analysis and their internal interaction relationships. The green color represents TFs. The purple color represents miRNAs. The blue color represents mRNAs. **(B)** miRNAs and proteins target the interaction regulatory network diagram. The blue color represents key pathways. The purple color represents miRNAs. The pink color represents proteins.

**Fig.S4 LPS induces acute lung injury in vivo.** An experimental acute lung injury model was induced by intratracheal instillation of LPS (10 mg/kg) into rats. These rats were sacrificed 24 hours after LPS administration for the following experiments. **(A-B)** Representative H&E staining of lung tissues and the histogram showed the lung tissue pathological damage score. Scale bar, 50 μm. **(C)** The concentrations of IL-1β, IL-6, and TNF-α in BALF were measured by ELISA. **(D)** The concentrations of IL-1β, IL-6, and TNF-α in serum were measured by ELISA. **(E)** Evans blue content in lung tissue. **(F)** The wet/dry weight ratio in lung tissue. **(G)** Observation of EV morphology by TEM; Scale bar: 100 nm. **(H)** NanoFCM of EV diameter. **(I)** Western blot analysis of the expression of EV markers (Alix, Tsg101, and CD9) and Non-EV markers (GM130, calnexin, and TIM23) in cells and EVs. Representative results from three independent experiments are shown (n = 6 rats/group). Data are presented as the mean ± SEM. ns, no significance, **p* < 0.05, ***p* < 0.01, ****p* < 0.001,*****p* < 0.0001.

**Supplementary Table 1** Sequences used in this study

| Gene | Forward (5′-3′ ) | Reverse (5′-3′ ) |
| --- | --- | --- |
| hsa-miR-30e-3p | CGCTTTCAGTCGGATGTTTACAGC |  |
| hsa-miR-99a-5p | CAACCCGTAGATCCGATCTTGTG |  |
| hsa-miR-122-5p | TGGAGTGTGACAATGGTGTTTG |  |
| hsa-miR-125b-5p | CTCCCTGAGACCCTAACTTGTGA |  |
| hsa-miR-150-5p | TCTCCCAACCCTTGTACCAGTG |  |
| hsa-miR-155-5p | CGCTTAATGCTAATCGTGATAGGGGTT |  |
| hsa-miR-223-3p | GCCTGTCAGTTTGTCAAATACCCCA |  |
| hsa-miR-378a-3p | ACTGGACTTGGAGTCAGAAGGC |  |
| U6 | GGAACGATACAGAGAAGATTAGC | TGGAACGCTTCACGAATTTGCG |
| IL-6 | AAAGAGTTGTGCAATGGCAATTCT | AAGTGCATCATCGTTGTTCATACA |
| IL-1β | AATCTCACAGCAGCATCTCGACAAG | TCCACGGGCAAGACATAGGTAGC |
| TNF-α | ATGGGCTCCCTCTCATCAGTTCC | CCTCCGCTTGGTGGTTTGCTAC |
| iNOS | AATGGTGGAGGTGCTGGAGGAG | GTCTGGAGAGGAGCTGATGGAGTAG |
| Mrc1 | TGGACAGACGGACGAGGAGTTC | GCCACCAATCACAACAACACAGTC |
| ACSL4 | CATATCGCTCTGTCACGCACTTC | GGCTGTCCTTCTTCCCAAACTTG |
| GPX4 | GCAGGAGCCAGGAAGTAATCAAG | ACAGTGGGTGGGCATCGTC |
| HO-1 | GGAAGAGGAGATAGAGCGAAACAAG | TGGCTGGTGTGTAAGGGATGG |
| SLC7A11 | CATCATCATCGGCACCGTCATC | TCCACAGGCAGACCAGAACAC |
| P62 | CCAGCACAGGCACAGAAGATAAGAG | TCCCACCGACTCCAAGGCTATC |
| LC3B | CAAGCCTTCTTCCTCCTGGTGAATG | AGTGCTGTCCCGAACGTCTCC |
| ATG5 | ATATGAAGGCACACCCCTGAAATGG | GTGATGTTCCAAGGCAGAGCTGAG |
| Beclin-1 | TCAAGATCCTGGACCGAGTGACC | CTCCTCTCCTGAGTTAGCCTCTTCC |
| β-actin | TACTGCCCTGGCTCCTAGCA | TGGACAGTGAGGCCAGGATAG |

**Supplementary Table 2** Antibodies used in this study

| Antibodies | Source | Identifier | Dilution ratio |
| --- | --- | --- | --- |
| LC3B | Abmart | T55992S | 1:1000 |
| MEF2C | Abcam | ab231859 | 1:1000 |
| YAP | CST | 4912 | 1:1000 |
| P-YAP | CST | 4911 | 1:1000 |
| Calnexin | Proteintech | 10427-2-AP | 1:5000 |
| GM130 | Proteintech | 11308-1-AP | 1:5000 |
| TIM23 | Proteintech | 11123-1-AP | 1:1000 |
| ALIX | Zen-bio | R23425 | 1:1000 |
| CD9 | Zen-bio | 380441 | 1:1000 |
| TSG101 | Zen-bio | 381538 | 1:1000 |
| ATG5 | Zen-bio | R23497 | 1:1000 |
| Beclin1 | Zen-bio | R22856 | 1:1000 |
| P62 | Zen-bio | 380612 | 1:1000 |
| ACSL4 | Zen-bio | R24265 | 1:1000 |
| HO-1 | Zen-bio | R24541 | 1:1000 |
| GPX4 | Zen-bio | 381958 | 1:1000 |
| LATS1 | Zen-bio | 252567 | 1:500 |
| β-actin | Zen-bio | 380624 | 1:10000 |

**Supplementary Table 3** Demographic and clinical details of patients with sepsis and septic shock

| Characteristics | All patients  N = 115 | Sepsis  N＝49 | Septic shock  N＝66 | *P-value* |
| --- | --- | --- | --- | --- |
| **Sociodemographic characteristics** | | | | |
| Age, years | 58 (47–68) | 57 (45–67) | 58 (47–68) | 0.524 |
| Male sex, n(%) | 86 (74.8) | 35 (71.4) | 51 (77.3) | 0.475 |
| **Comorbidities, n(%)** |  |  |  |  |
| Arterial hypertension | 43 (37.4) | 17 (34.7) | 26 (39.4) | 0.606 |
| Diabetes mellitus | 18 (15.7) | 8 (16.3) | 10 (15.2) | 0.864 |
| Cerebrovascular disease | 7 (6.1) | 2 (4.1) | 5 (7.6) | 0.438 |
| **Source of sepsis, n(%)** |  |  |  |  |
| Lung | 82 (71.3) | 30 (61.2) | 52 (78.8) | 0.039 |
| Abdominal | 20 (17.4) | 7 (14.3) | 13 (19.7) | 0.449 |
| Skin and soft tissue | 7 (6.1) | 1 (2.04) | 6 (9.1) | 0.118 |
| Other | 29 (25.2) | 20 (40.8) | 9 (13.6) | 0.001 |
| **ICU admission** |  |  |  |  |
| MAP (mmHg) | 84.8 ± 21.6 | 89.9 ± 21.8 | 81 ± 21 | 0.027 |
| WBC (10^9^/L) | 13.8 ± 6.5 | 13.3 ± 6.3 | 14.2 ± 6.8 | 0.384 |
| HGB (g/L) | 99 ± 35 | 91.8 ± 33.7 | 104.3 ± 34.8 | 0.039 |
| PLT (10^9^/L) | 204 (103–288) | 174 (78.5-267.3) | 213 (116–295) | 0.930 |
| PCT (ng/mL) | 2.23 (0.5–14.5) | 1.4 (0.4-9) | 3.9 (0.7–19) | 0.036 |
| CRP (mg/L) | 88 (24.3–145) | 75 (17–133) | 96.4 (28-163.1) | 0.130 |
| proBNP (pg/mL) | 2640 (523-10525) | 2653 (858–8505) | 2628 (407-11023) | 0.355 |
| PT (s) | 13.4 (11.9–15.6) | 13.5 (12–16) | 13.1 (12-15.6) | 0.673 |
| APTT ( s) | 32.4 (29–36) | 32.2 (28.5–35.6) | 32.8 (28.6–37) | 0.416 |
| FIB (g/L) | 4.35 ± 1.67 | 4.2 ± 1.5 | 4.4 ± 1.8 | 0.481 |
| TB (umol/L) | 14.4 (9.5–25.1) | 13.7 (8.4–22.8) | 15.8 (9.7–28.7) | 0.141 |
| Albumin (g/L) | 31.6 ± 5.3 | 32 ± 5.2 | 31.2 ± 5.3 | 0.327 |
| Scr (umol/L) | 119 (79–220) | 107 (79.3–192) | 131 (77–255) | 0.088 |
| Lactate (mmol/L) | 2.02 (1.4–3.8) | 1.5 (1.2–2.5) | 2.6 (1.7–4.6) | < 0.001 |
| Use of mechanical ventilation, n(%) | 92 (80) | 39 (79.6) | 53 (80.3) | 0.925 |
| Mechanical ventilation duration (days) | 2.5 (1–8) | 1.3 (0.3-8) | 3 (1-7.7) | 0.722 |
| PaO_2_/FiO_2_ (mmHg) | 230 (164–336) | 225 (163–380) | 232 (164–291) | 0.903 |
| SOFA score | 8 (6–10) | 8 (5.3–9.5) | 8 (7–10) | 0.013 |
| APACHE II score | 20 (17-24.3) | 20 (17-22.8) | 22 (16.8–26) | 0.084 |
| **ICU Stay** |  |  |  |  |
| ICU stay (days) | 4 (1-10.3) | 3 (1-11.8) | 5 (2–10) | 0.895 |
| 28-day mortality, n (%) | 55 (47.8) | 21 (42.9) | 34 (51.5) | 0.358 |

**Abreviations:** APTT, Activated partial thromboplastin time; APACHE, Acute physiology and chronic health evaluation; CRP, C-reactive protein; FIB, Fibrinogen; ICU = Intensive care unit; MAP, mean arterial pressure; PCT, Procalcitonin; PT, Prothrombin time; pro-BNP, pro-brain natriuretic peptide; PaO_2_/FiO_2_, Ratio of partial pressure of arterial oxygen to the fraction of inspired oxygen; TB, Total bilirubin; Scr, Serum creatinine; SOFA, Sequential organ failure assessment score; WBC, White blood cells.

**Supplementary Table 4** Demographic and clinical details of survivors and non-survivors

| Characteristics | Survivors  N = 60 | Non-survivors  N = 55 | *P-value* |
| --- | --- | --- | --- |
| **Sociodemographic characteristics** | | | |
| Age, years | 59 (43.8–68.3) | 57.5 (52–68) | 0.962 |
| Male sex, n(%) | 44 (73.3) | 42 (76.4) | 0.709 |
| **Comorbidities, n(%)** |  |  |  |
| Arterial hypertension | 24 (40) | 19 (34.5) | 0.546 |
| Diabetes mellitus | 11 (18.3) | 7 (12.7) | 0.409 |
| Cerebrovascular disease | 4 (6.7) | 3 (5.5) | 0.786 |
| **Source of sepsis, n(%)** |  |  |  |
| Lung | 42 (70) | 40 (72.7) | 0.747 |
| Abdominal | 12 (20) | 8 (14.5) | 0.441 |
| Skin and soft tissue | 2 (3.3) | 5 (9.1) | 0.197 |
| Other | 15 (25) | 14 (25.5) | 0.955 |
| **ICU admission** |  |  |  |
| MAP (mmHg) | 84.2 ± 19.9 | 85.5 ± 23.7 | 0.364 |
| WBC (10^9^/L) | 13 ± 6.2 | 14.7 ± 6.9 | 0.082 |
| HGB (g/L) | 99 ± 36.2 | 98.9 ± 33.4 | 0.835 |
| PLT (10^9^/L) | 216.5 (138.5-295.3) | 167.1 (66.3-258.5) | 0.065 |
| PCT (ng/mL) | 1.83 (0.41–7.97) | 2.6 (0.58–19.8) | 0.165 |
| CRP (mg/L) | 83.6 (19.1-143.3) | 90.9 (26.2-166.4) | 0.439 |
| proBNP (pg/mL) | 1795 (407–9300) | 3417 (769-11369) | 0.696 |
| PT (s) | 13.2 (11.7–16) | 13.5 (12-15.3) | 0.438 |
| APTT ( s) | 32 (29–35) | 33 (28.5–37.7) | 0.149 |
| FIB (g/L) | 4.5 ± 1.5 | 4.2 ± 1.9 | 0.462 |
| TB (umol/L) | 15.8 (10.2–29.9) | 12.9 (8.1–22.1) | 0.319 |
| Albumin (g/L) | 32.2 ± 6 | 30.8 ± 4.2 | 0.113 |
| Scr (umol/L) | 113 (76–276) | 120 (82–193) | 0.486 |
| Lactate (mmol/L) | 1.7 (1.3–2.6) | 3 (1.5–4.9) | 0.007 |
| Use of mechanical ventilation, n(%) | 48 (80) | 44 (80) | 1.000 |
| Mechanical ventilation duration (days) | 3 (0.56-10) | 2 (1-6.8) | 0.172 |
| PaO_2_/FiO_2_ (mmHg) | 255 (194–346) | 200 (123–300) | 0.023 |
| SOFA score | 7 (5–8) | 10 (8-11.8) | < 0.001 |
| APACHE II score | 17 (14-19.3) | 24 (22.3–26) | < 0.001 |
| **ICU stay (days)** | 6 (2–12) | 2.5 (1–8) | < 0.001 |

**Abreviations:** APTT, Activated partial thromboplastin time; APACHE, Acute physiology and chronic health evaluation; CRP, C-reactive protein; FIB, Fibrinogen; ICU = Intensive care unit; MAP, mean arterial pressure; PCT, Procalcitonin; PT, Prothrombin time; pro-BNP, pro-brain natriuretic peptide; PaO_2_/FiO_2_, Ratio of partial pressure of arterial oxygen to the fraction of inspired oxygen; TB, Total bilirubin; Scr, Serum creatinine; SOFA, Sequential organ failure assessment score; WBC, White blood cells.

**Supplementary Table 5** Demographic and clinical details of survivors and non-survivors with septic ARDS

| Characteristics | Survivors  N = 27 | Non-survivors  N = 37 | *P-value* |
| --- | --- | --- | --- |
| **Sociodemographic characteristics** | | | |
| Age, years | 59 (40–68) | 60 (50–68) | 0.644 |
| Male sex, n(%) | 21 (77.8) | 29 (78.4) | 0.954 |
| **Comorbidities, n(%)** |  |  |  |
| Arterial hypertension | 8 (29.6) | 13 (35.1) | 0.643 |
| Diabetes mellitus | 5 (18.5) | 3 (8.1) | 0.214 |
| Cerebrovascular disease | 2 (7.4) | 3 (8.1) | 0.918 |
| **Source of sepsis, n(%)** |  |  |  |
| Lung | 27 (100) | 37 (100) | 1.000 |
| Abdominal | 8 (29.6) | 5 (13.5) | 0.114 |
| Skin and soft tissue | 1 (3.7) | 4 (10.8) | 0.295 |
| Other | 2 (7.4) | 0 (0) | 0.093 |
| **ICU admission** |  |  |  |
| MAP (mmHg) | 81.2 ± 20.2 | 85.5 ± 25.2 | 0.924 |
| WBC (10^9^/L) | 13.4 ± 7.4 | 15 ± 7.5 | 0.235 |
| HGB (g/L) | 102.4 ± 40.7 | 105.1 ± 32.7 | 0.615 |
| PLT (10^9^/L) | 235 (147–295) | 182 (72–274) | 0.242 |
| PCT (ng/mL) | 3. 8 (0.7–23.1) | 2.1 (0.4–17.3) | 0.563 |
| CRP (mg/L) | 114.6 (45.1-171.4) | 78.5 (26.7-149.5) | 0.293 |
| proBNP (pg/mL) | 3015 (1403–8935) | 2264 (746–7674) | 0.343 |
| PT (s) | 13.8 (12.2–16.1) | 13.4 (12-15.6) | 0.873 |
| APTT ( s) | 31 (27.6–35.2) | 32.8 (27.8–37.5) | 0.263 |
| FIB (g/L) | 4.3 ± 1.4 | 4.1 ± 1.9 | 0.683 |
| TB (umol/L) | 18 (10.3–31) | 12.8 (7.6–23.5) | 0.155 |
| Albumin (g/L) | 29.7 (25.5–33.8) | 30.6 (27.5–33.1) | 0.876 |
| Scr (umol/L) | 177 (100–424) | 112 (82–176) | 0.253 |
| Lactate (mmol/L) | 2.2 (1.5–3.7) | 3.4 (1.4–5.4) | 0.187 |
| Use of mechanical ventilation, n(%) | 23 (85.2) | 29 (78.4) | 0.491 |
| Mechanical ventilation duration (days) | 5.7 (2–10) | 3 (1-7.3) | 0.032 |
| PaO_2_/FiO_2_ (mmHg) | 187.4 ± 58.7 | 164 ± 67.3 | 0.255 |
| SOFA score | 7 (6–8) | 10 (8–13) | < 0.001 |
| APACHE II score | 17.7 ± 3.3 | 24.2 ± 2.9 | < 0.001 |
| **ICU stay (days)** | 6 (3–12) | 3 (1–8) | 0.005 |

**Abreviations:** APTT, Activated partial thromboplastin time; APACHE, Acute physiology and chronic health evaluation; CRP, C-reactive protein; FIB, Fibrinogen; ICU = Intensive care unit; MAP, mean arterial pressure; PCT, Procalcitonin; PT, Prothrombin time; pro-BNP, pro-brain natriuretic peptide; PaO_2_/FiO_2_, Ratio of partial pressure of arterial oxygen to the fraction of inspired oxygen; TB, Total bilirubin; Scr, Serum creatinine; SOFA, Sequential organ failure assessment score; WBC, White blood cells.

**Supplementary Table 6** Logistic regression in patients with septic shock

| Risk Factors | β | SE | Wald X^2^ | *P* | OR | 95%CI | |
| --- | --- | --- | --- | --- | --- | --- | --- |
|  |  |  |  |  |  | Lower | Higher |
| LCN2 | 0.002 | 0.131 | < 0.001 | 0.985 | 1.002 | 0.776 | 1.295 |
| OLFM4 | 0.006 | 0.002 | 14.897 | < 0.001 | 1.006 | 1.003 | 1.010 |
| miR-122-5p | 0.395 | 0.191 | 4.290 | 0.038 | 1.485 | 1.021 | 2.158 |
| miR-125b-5p | 0.548 | 0.230 | 5.655 | 0.017 | 1.729 | 1.101 | 2.715 |
| miR-223-3p | -0.065 | 0.126 | 0.272 | 0.602 | 0.937 | 0.732 | 1.198 |

**Abreviations:** CI = confidence interval; OR = odds ratio.

**Supplementary Table 7** Logistic regression for predicting the prognosis of patients with sepsis

| Risk Factors | β | SE | Wald X^2^ | *P* | OR | 95%CI | |
| --- | --- | --- | --- | --- | --- | --- | --- |
|  |  |  |  |  |  | Lower | Higher |
| LCN2 | 0.282 | 0.135 | 4.396 | 0.036 | 1.326 | 1.019 | 1.726 |
| OLFM4 | 0.002 | 0.001 | 5.196 | 0.023 | 1.002 | 1.000 | 1.005 |
| miR-122-5p | 0.486 | 0.182 | 7.110 | 0.008 | 1.625 | 1.137 | 2.322 |
| miR-125b-5p | 0.336 | 0.204 | 2.732 | 0.098 | 1.400 | 0.939 | 2.086 |
| miR-223-3p | -0.163 | 0.120 | 1.852 | 0.174 | 0.850 | 0.672 | 1.074 |

**Abreviations:** CI = confidence interval; OR = odds ratio.

**Supplementary Table 8** Logistic regression for predicting the prognosis of patients with septic ARDS

| Risk Factors | β | SE | Wald X^2^ | *P* | OR | 95%CI | |
| --- | --- | --- | --- | --- | --- | --- | --- |
|  |  |  |  |  |  | Lower | Higher |
| miR-122-5p | 0.635 | 0.221 | 8.228 | 0.004 | 1.886 | 1.223 | 2.910 |
| miR-223-3p | 0.028 | 0.143 | 0.038 | 0.845 | 1.028 | 0.777 | 1.361 |
| LCN2 | 0.304 | 0.178 | 2.916 | 0.088 | 1.355 | 0.956 | 1.920 |
| OLFM4 | < 0.001 | 0.002 | < 0.001 | 0.978 | 1.000 | 0.997 | 1.003 |

**Abreviations:** CI = confidence interval; OR = odds ratio.
